# ‘Aggregation-Induced Emission’ Active Mono-Cyclometalated Iridium(III) Complex Mediated Efficient Vapor-Phase Detection of Dichloromethane

**DOI:** 10.3390/molecules27010202

**Published:** 2021-12-29

**Authors:** Pramod C. Raichure, Vishal Kachwal, Inamur Rahaman Laskar

**Affiliations:** 1 Department of Chemistry, Birla Institute of Technology and Science, Pilani Campus, Pilani 333031, Rajasthan, India; raichurepramod@gmail.com (P.C.R.); vishkachwal4@gmail.com (V.K.); 2 Department of Engineering Science, University of Oxford, Oxford OX1 3PJ, UK

**Keywords:** aggregation-induced emission, dichloromethane, vapor phase, cost-effective

## Abstract

Selective vapor-phase detection of dichloromethane (DCM) is a challenge, it being a well-known hazardous volatile organic solvent in trace amounts. With this in mind, we have developed an ‘Aggregation-induced Emission’ (AIE) active mono-cyclometalated iridium(III)-based (M1) probe molecule, which detects DCM sensitively and selectively in vapor phase with a response time <30 s. It reveals a turn-on emission (non-emissive to intense yellow) on exposing DCM vapor directly to the solid M1. The recorded detection limit is 4.9 ppm for DCM vapor with pristine M1. The mechanism of DCM detection was explored. Moreover, the detection of DCM vapor by M1 was extended with a low-cost filter paper as the substrate. The DCM is weakly bound with the probe and can be removed with a mild treatment, so, notably, the probe can be reused.

## 1. Introduction

The development of a selective and sensitive probe for hazardous volatile organic compounds (VOCs) is becoming a thrust area in the present day [1]. Most solvents are often used for various purposes to solve our daily problems. Dichloromethane (DCM) is commonly used as a solvent in laboratories and as a paint remover in industry; it is also used as an aerosol propellant, degreaser agent, room deodorant, herbicide, and insecticide [2,3]. Nowadays, it is not unknown that DCM inhalation causes severe health problems. A recent case of DCM injection injury in the finger shows the gravity of the toxicity of DCM [4]. The excessive usage and the high volatility of DCM result in significant contamination of water and air [5]. In general, the detection of DCM relies on gas chromatography [6,7], mass spectroscopy [8], or bioluminescent markers [9]. All these techniques require considerable time, high cost, and significant effort. However, reports on fluorescent-based sensing materials show sensitive detection of many organic solvents, including chloroform, DCM, methanol, acetone, tetrahydrofuran (THF), etc. Still, there is a concern over lack of selectivity, fast response, or the need for specific kinds of substrates [10,11,12,13,14]. Moreover, a more reliable, selective, and sensitive technique is needed in a real-time application.

Solid-state organic-based luminescent materials show a wide range of applications such as organic light-emitting diodes (OLED), solar cells, metal ion sensing, stimuli-responsive materials, bacterial imaging, etc. [15,16,17,18,19]. However, traditional organic luminophores exhibit emission quenching in the aggregated state, called ‘Aggregation-caused Quenching’ (ACQ), which limits their applications. Tang et al. first proposed a new concept called ‘Aggregation-induced Emission’ (AIE) in 2001 [20]. The luminescence of AIE active molecules in the solid phase is stronger than their solution or dispersed state. The AIE molecules emerged as a novel advanced material due to their excellent performance in various fields [21].

Recently, the research has been focused on fluorescence-based techniques for detecting volatile organic solvents, as the technique is more reliable and reduces the response time [22,23]. Liang et al. first explored quick and easy turn-on fluorescence sensing of DCM in a liquid state only, where Ln(III) complexes were synthesized through Schiff base-exchange reaction [24]. In 2020, Lang et al. published an article describing the host–guest chemistry by utilizing the metal–organic framework materials synthesized by CuI and tripyridyl phosphine for sensing chlorinated solvents (CH_2_Cl_2_, CHCl_3_, and chlorobenzene) [25]. The technique is based on the host–guest chemistry, i.e., lock the guest solvent molecules in MOF, which induces luminescence enhancement. The MOF material is non-emissive in the absence of solvent molecules due to internal vibrations. It shows turn-on emission after restrictions of vibrations in the presence of solvent molecules. Here, the response time was less (<1 s), and also recyclability was high, but the materials were not selective to the DCM. It can also sense chloroform, carbon tetrachloride, and emissions observed at the same wavelength (580 nm). Recently, Li et al. developed an ESIPT (excited-state intramolecular proton transfer) active N-linked salicylaldehyde Schiff base organic compound to detect the Cs^+^ and DCM with turn-off emission on the ligand to metal charge transfer (LMCT) process [26]. The detection limit in the case of DCM was 0.37% (*v*/*v*, 3700 ppm) in a solution state. All these studies reveal that a significant insufficiency exists in all the developed luminescent-based probes for detecting DCM.

Herein, we reported a new AIE active mono-cyclometalated Ir(III) complex (M1) for selective and sensitive detection of the DCM vapors. The synthesized M1 complex is non-emissive, but it shows light-on strong yellow emission upon interaction with DCM. The DCM vapors can be sensed on M1 impregnated filter paper as well as on thin film. The recorded detection limit for DCM vapor with M1 pristine is 4.9 ppm. To the best of our knowledge, it is the lowest limit of detection (LOD) obtained for a reusable, selective, and sensitive DCM detection with pristine probe till now [27].

## 2. Results and Discussion

**Scheme 1:**
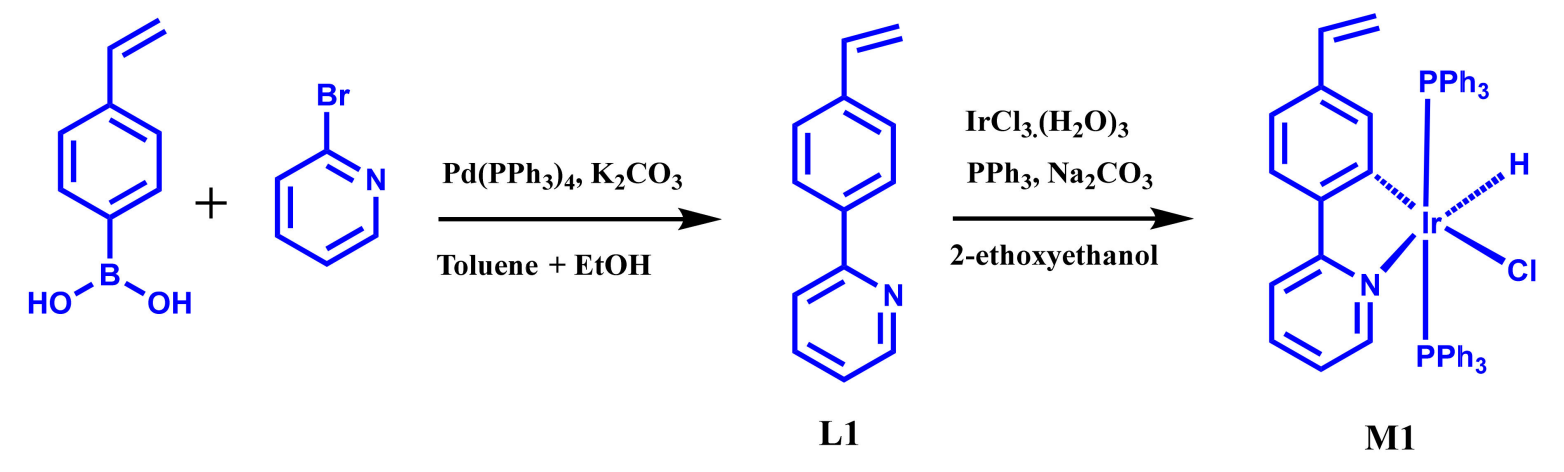
Synthesis of the ligand L1 and iridium(III) complex M1.

The iridium(III)-based heavy-metal complex, M1 synthesized from ligand L1 (Scheme 1). The synthetic procedure for L1 and M1 is reported in the Supplementary Material. The synthesized compounds L1 and M1 are characterized by ^1^H, ^13^C NMR analysis (Figures S1–S4, †ESI). In ^1^H-NMR (400 MHz, Chloroform-*d*), the observed peak at δ −16.82 (s, 1H) corresponds to iridium(III) hydride. In ^31^P-NMR (162 MHz, Chloroform-*d*), the observed singlet peak at δ 9.01 relates the phosphorous in PPh_3_ ligand of M1 (Figure S5, †ESI). In HRMS spectrum, the observed mass corresponds to the calculated mass: [M-Cl]^+^: *m*/*z* = 898.21; found: [M-Cl]^+^: *m*/*z* = 898.23 (Figure S6, †ESI).

**Figure 1:**
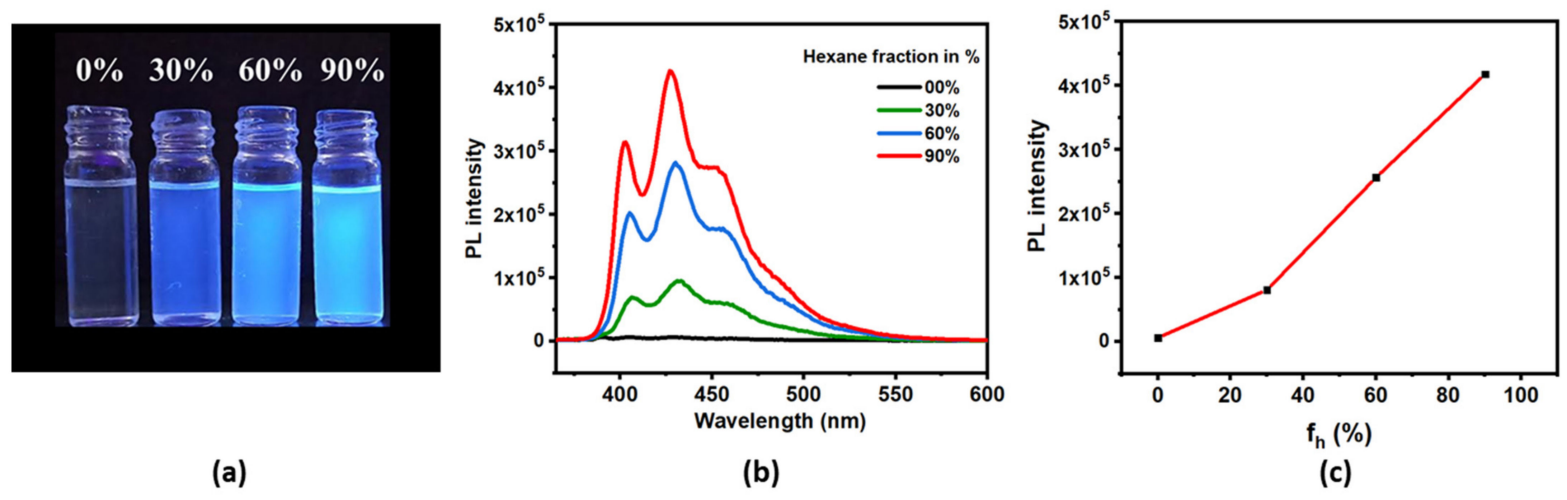
(**a**) Photograph of M1 in THF: hexane mixtures of various hexane fractions (f_h_) in percentage (%) under UV lamp (365 nm); (**b**) PL spectra for the same under excitation of 360 nm wavelength; (**c**) Line plot for the changes in PL intensity for M1 with gradual increasing hexane percentage (f_h_) in THF: hexane mixture.

Here, the synthesized M1 is tested as an AIE-active compound. The AIE experiment for M1 is carried out by taking a mixture of THF and hexane solvents and preparing the mixture, as reported in supporting information (Figure S8, †ESI). The photoluminescence (PL) spectra of all the prepared solutions were recorded, and it indicates the PL intensity steadily increases with increasing hexane fractions. It can be explained by the AIE effect (Figure 1) [28]. The formation of aggregates with increasing concentration of hexane in THF was analyzed by dynamic light scattering (DLS) particle size distribution experiment. The observed particle size for 0% hexane percentage (f_h_) is 52.3 nm (with PDI 0.2) and for 90% hexane percentage (f_h_) is 170.5 nm (with PDI 1.1) (Figure S7, †ESI), which supports the formation of aggregates.

The reason behind the AIE activity has often been described as the restriction of intramolecular rotation (RIR) of the peripheral ring in the aggregated state, which reduces the non-radiative decay and increases the radiative pathway resulting in emission enhancement [28]. Here, the AIE effect is speculated due to the restricted intramolecular rotation (RIR) mechanism. To prove the RIR effect at the cause of AIE, a viscous solvent poly(ethylene)glycol (PEG) was chosen, and the emission spectra of the solutions prepared with gradual increasing concentration of PEG into THF (gradual variation of viscosity of the medium) were studied (Figure S8, †ESI). It was observed that PL intensity increases with the gradual addition of PEG in THF: PEG mixture supporting the RIR mechanism.

**Figure 2:**
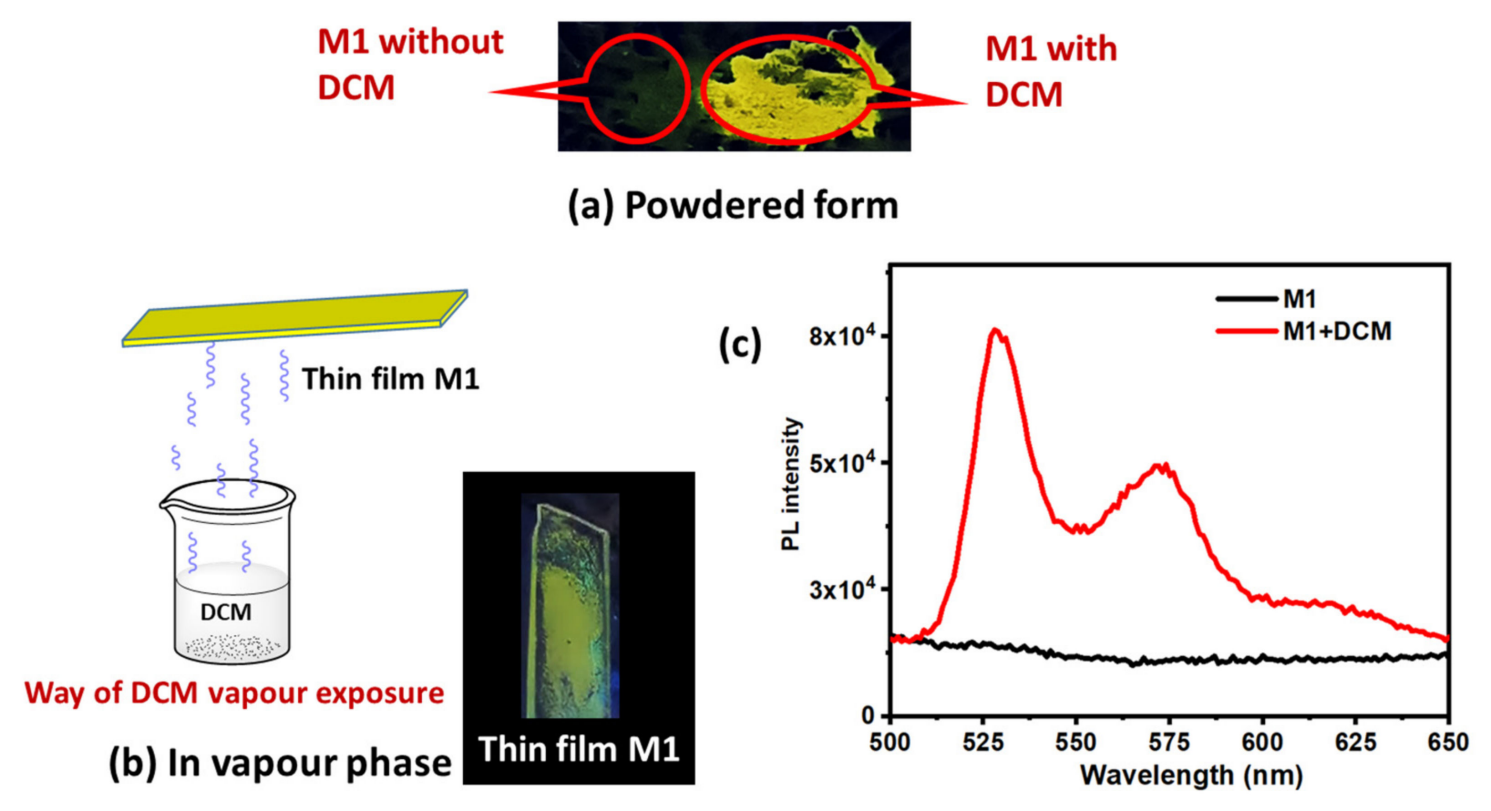
Images for DCM sensing: (**a**) in powdered form, (**b**) in the vapor phase with a representation of the way of exposing M1 thin film to DCM vapors (drawn) (left); actual image of emission after exposure to DCM vapor (right), (**c**) PL spectra for powdered M1 and M1 with DCM (wavelength: excitation = 360 nm, emission = 530 nm).

**Figure 3:**
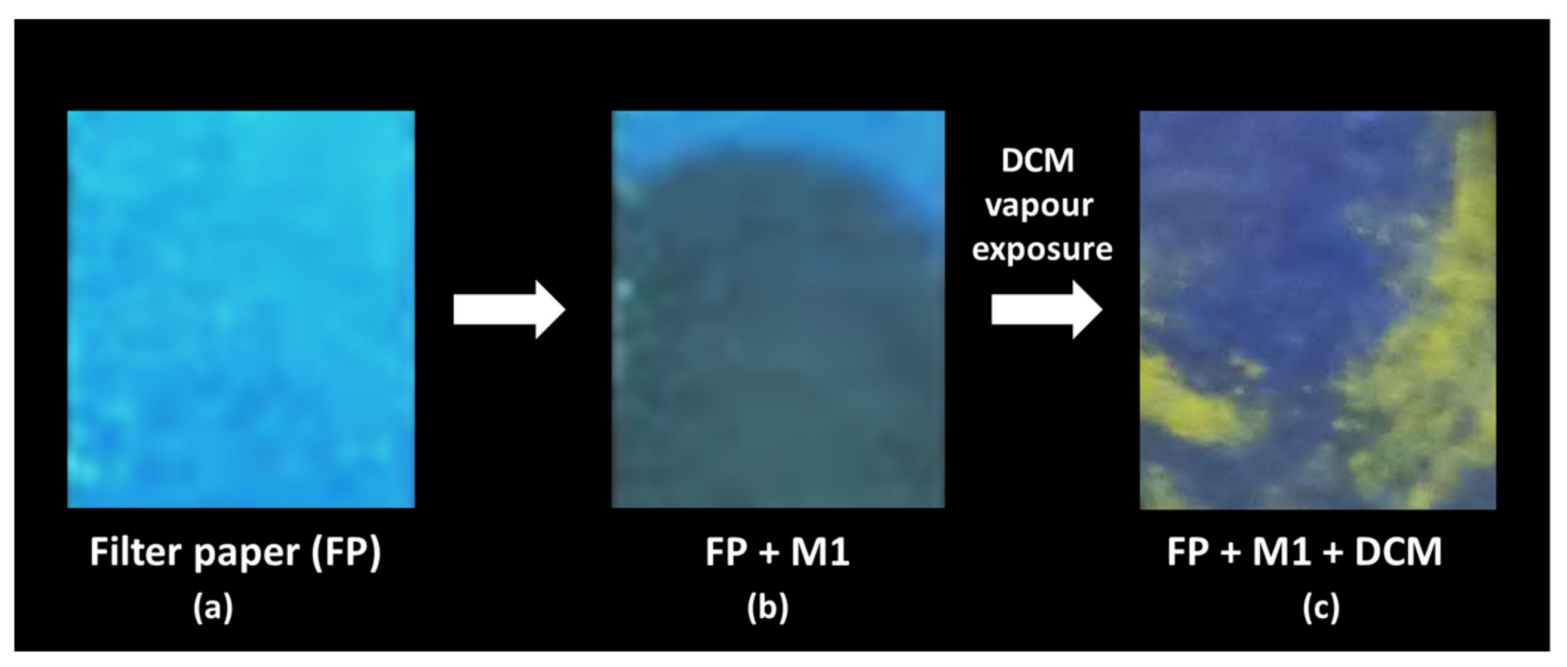
Images of filter paper (FP) under UV-visible lamp (365 nm): (**a**) filter paper (FP) only; (**b**) filter paper (impregnated with a solution of M1 in toluene) without DCM vapor; and (**c**) filter paper (impregnated with M1) in the presence of DCM vapors (turn on emission can be observed in the presence of DCM vapors).

The DCM detection tested with the probe M1 in pristine form (in powdered and thin-film also). The thin film of M1 is made on a glass substrate by a drop-casting method. It was observed that non-emissive M1 shows turn-on yellow emission at 530 nm by putting a drop of DCM on powdered M1 and for thin-film upon 30 s exposure of DCM vapor generated by setting the temperature at 33 °C (Figure 2). The probe M1 could also detect the DCM on impregnated filter paper with M1 (Figure 3).

**Figure 4:**
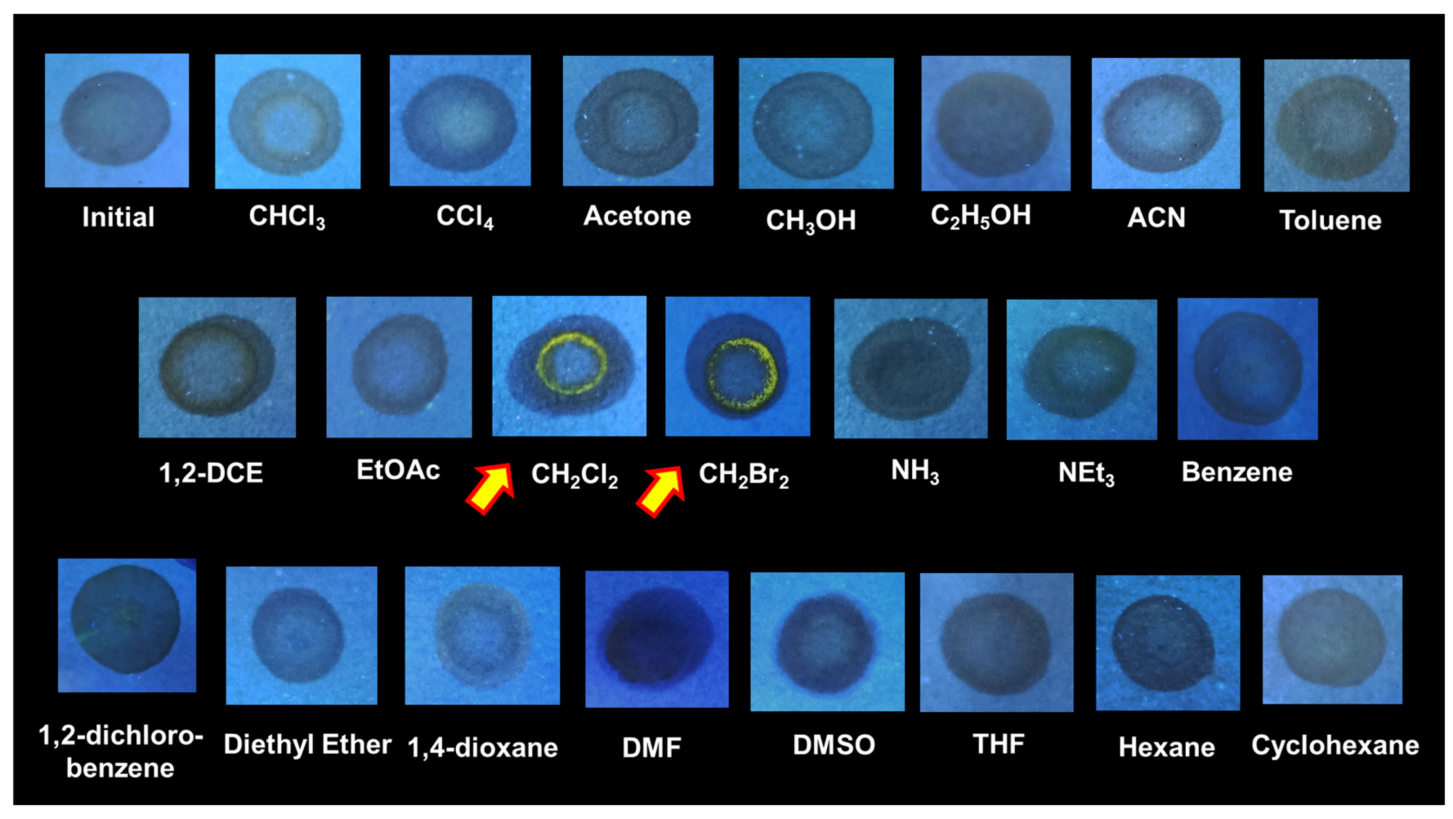
Photograph of selectivity test of M1 with the various solvents chloroform (CHCl_3_), carbon tetrachloride (CCl_4_), acetone, methanol (CH_3_OH), ethanol (C_2_H_5_OH), acetonitrile (ACN), toluene, 1,2-dichloroethane (1,2-DCE), ethyl acetate (EtOAc), dichloromethane (CH_2_Cl_2_), dibromomethane (CH_2_Br_2_), ammonia (NH_3_), triethylamine (NEt_3_), benzene, 1,2-dichlorobenzene, diethyl ether, 1,4-dioxane, N, N-Dimethylformamide (DMF), dimethyl sulfoxide (DMSO), tetrahydrofuran (THF), hexane, and cyclohexane on Whatman No 1 filter paper under UV lamp (365 nm) (The arrows indicate the turn-on emission response with dichloromethane and dibromomethane only).

To check the sensing selectivity of the M1 complex, it was screened with different solvents, which are generally used in industries and laboratories. For this experiment, the solution of M1 probe was prepared (1 mg M1 in 1 mL toluene). Then, equal amount (20 µL) drop of this prepared probe solution was placed on each of 24 small strips of Whatman No 1 filter paper by the drop-casting technique. After which, the filter paper strips containing M1 were annealed under the oven for about one hour at 70 °C. Various solvents were dropped on different annealed paper strips and observed emission, under a UV-visible lamp (Figure 4). The M1 shows turn-on yellow emission in the presence of DCM and its analogue dibromomethane (DBM) only, while not with other solvents (vide supra). This experiment clearly shows that the M1 selectively detects the DCM and DBM (Figure 4).

The M1 probe showed similar emission spectra while testing with DCM and DBM separately (λ_exi_, 360 nm) (Figure S10, †ESI). DBM is a high boiling solvent (97 °C), rarely used in laboratories and industries compared to its analogue DCM. All the sensing studies were performed with DCM as focal attention. After screening with different solvents, the selectivity of the probe M1 towards DCM was tested successfully.

**Figure 5:**
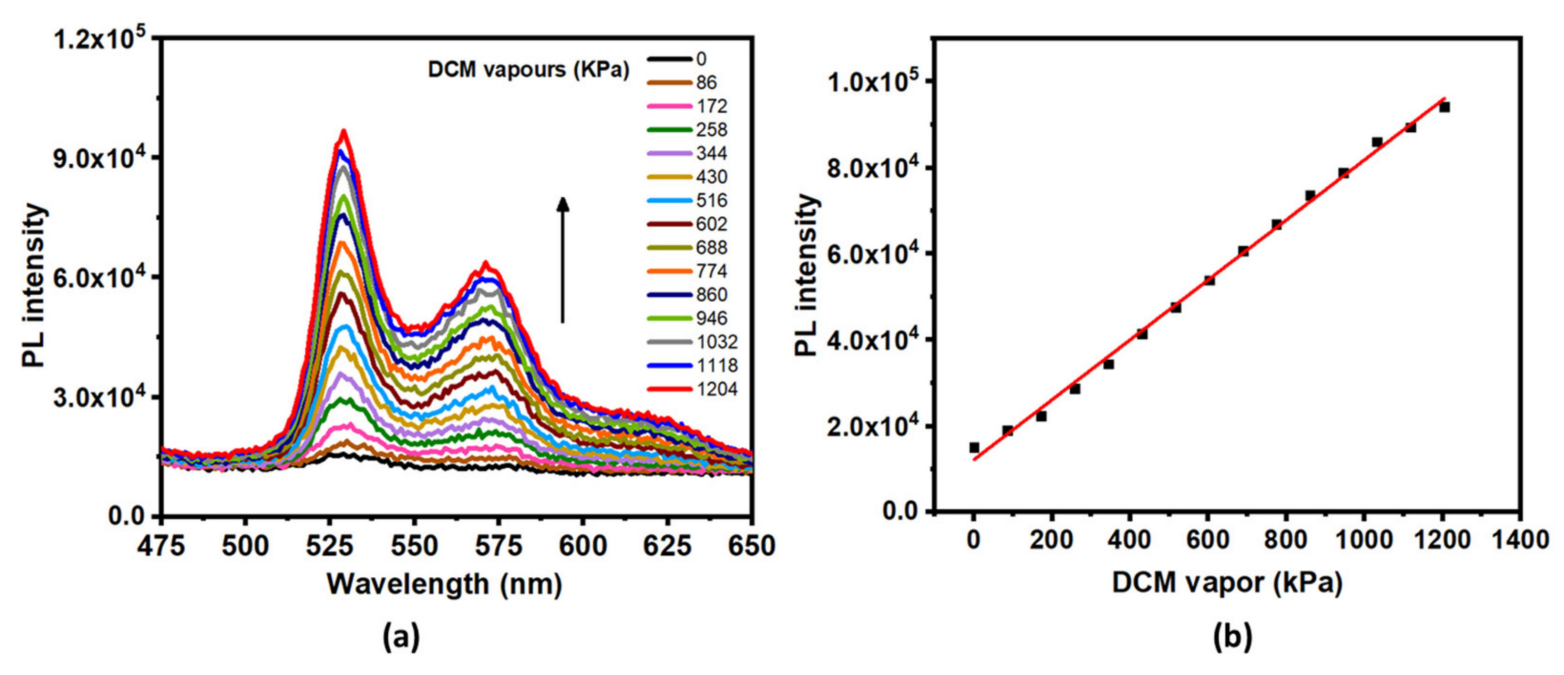
(**a**) PL spectra of M1 (powdered in solid sample holder) with gradually increasing DCM vapor concentration excited at 360 nm, (**b**) Plot between PL intensity of M1 vs. DCM vapor concentration (Pearson’s r value = 0.99).

The controlled experiment was performed to check DCM detection in vapor phase by generating a saturated DCM vapor in a closed pack container at a constant temperature, 33 °C (Figure S11, †ESI). To eliminate all the possibilities for the leakage of the DCM vapor, the container with the outlet was tightly air-packed. The M1 was taken as a powdered form in the solid sample holder. The sample holder was exposed to the DCM vapor at the outlet. Emission spectra were recorded by irradiating the solid sample holder directly under 360 nm excitation (Figure 5). For each reading, the solvent was heated to 33 °C for two minutes to generate the saturated vapors of DCM and M1 sample holder exposed to the DCM vapor for 30 s. The saturated vapor pressure of DCM at 33 °C is 86 kPa [29]. Thus, 86 kPa vapor of DCM was exposed to probe M1 for each reading. The emission intensity gradually increased for each exposure of DCM vapor. A linear relationship can be observed between the emission intensity of M1 and DCM vapor concentration from 0 to 1204 kPa (Figure 5). The obtained detection limit was 4.9 ppm (0.000497 kPa) (Figure S12, †ESI) in the vapor state, which is much less than the saturated vapor pressure of DCM at 33 °C (86 kPa). The LOD also crosses the setting inhalation limit (75 to 100 ppm for an hour inhalation) [30].

The probe M1 can be recycled. The powdered form of M1 treated with DCM vapor was kept in the open air. It was observed that the emission intensity of the probe decreased gradually and returned to pristine form on keeping the powder at 25 °C for 40 min. The rate of desorption of DCM is accelerated by heating the sample at 80 °C continuously for 30 s.

**Figure 6:**
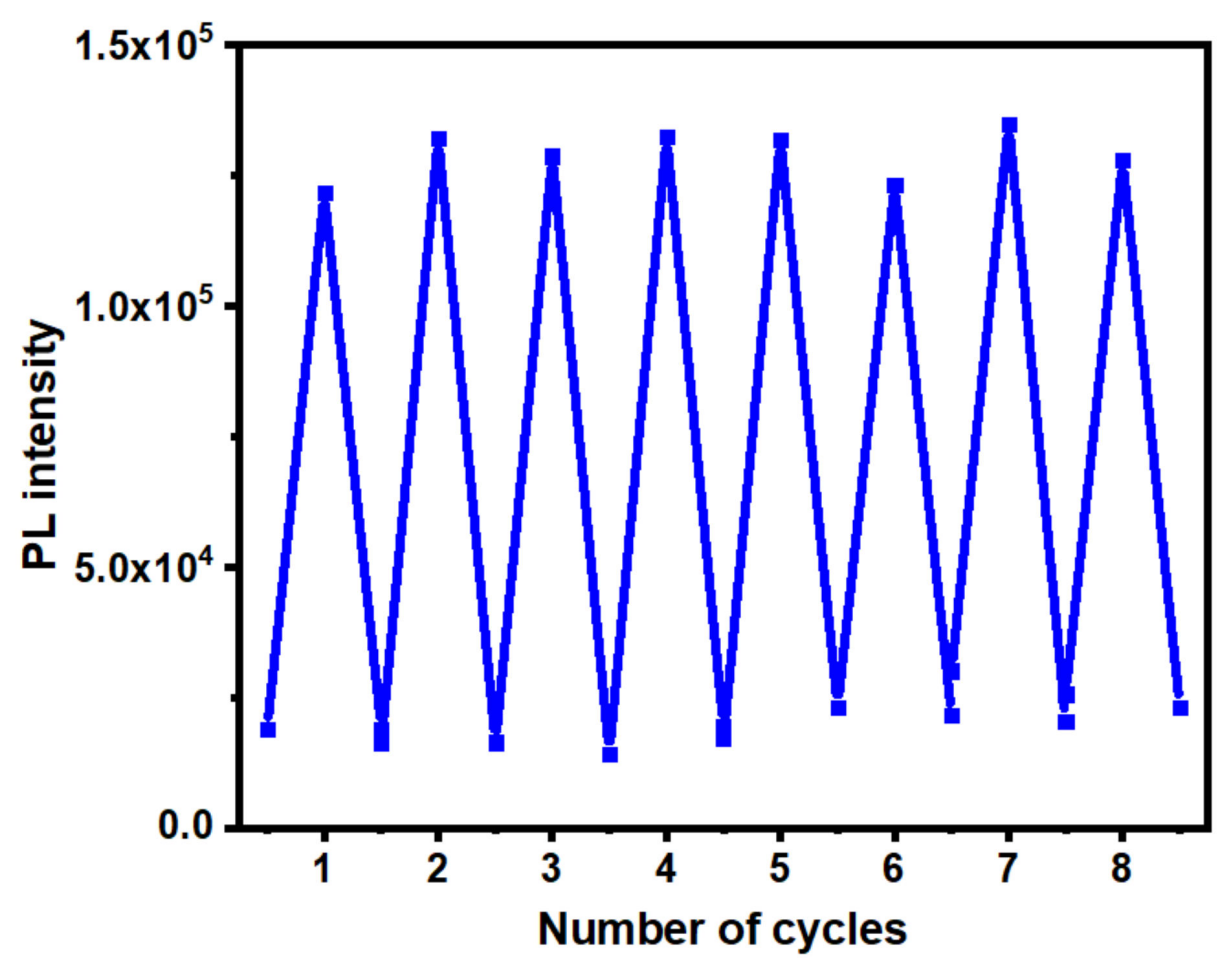
Recyclability study of M1 with DCM vapors. The emission of M1 after being exposed to DCM vapor returns to its original intensity by heating it at 80 °C continuously for 30 s.

The experiment was repeated several times to check the stability and reusability of the probe in the vapor phase (Figure 6). Even in the seventh cycle, the emission intensity almost remains the same. Hence, the same probe can be reused again for further sensing applications.

**Figure 7:**
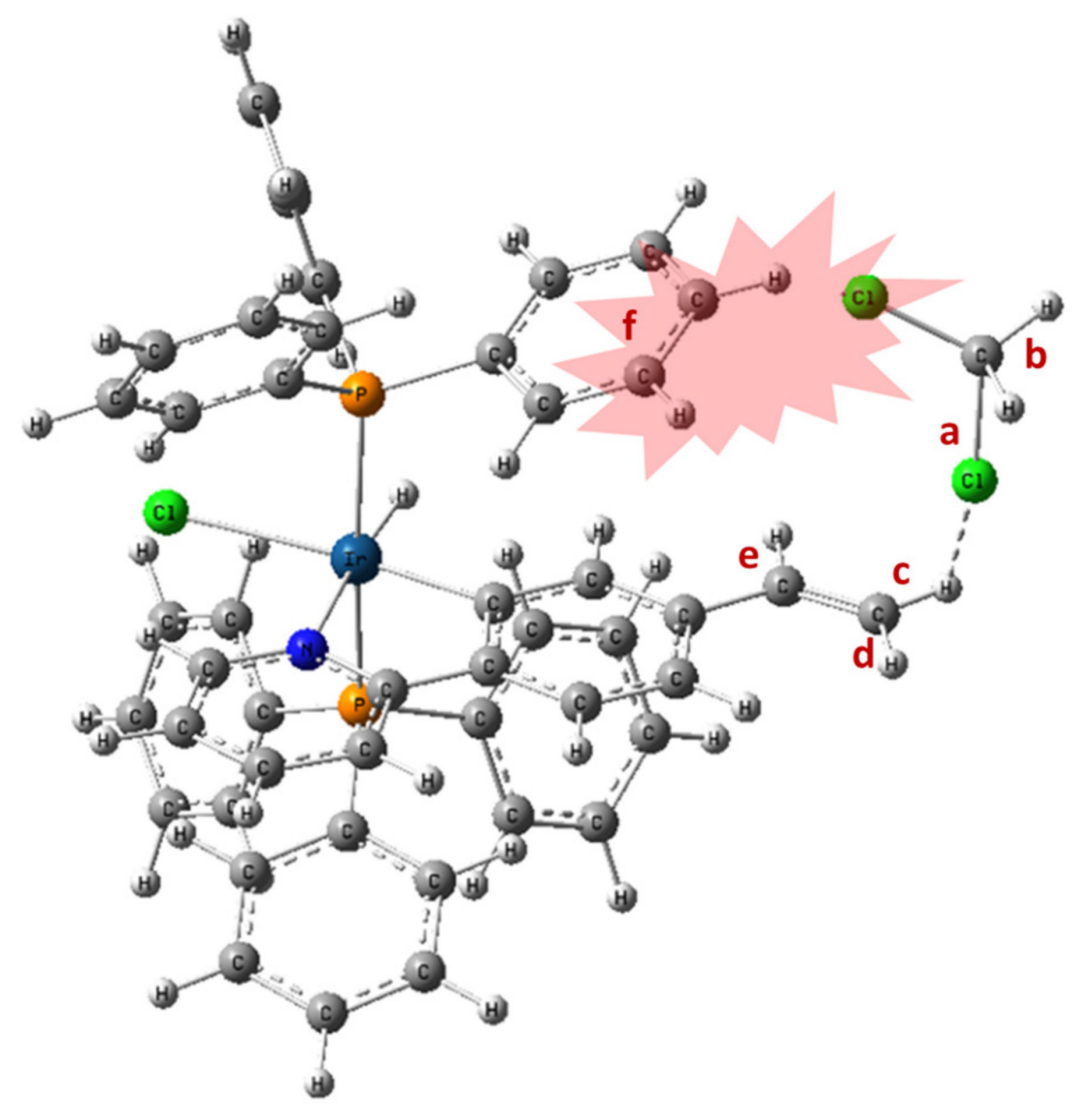
Schematic representative structural model of interaction of DCM with M1 labeling with characteristic bonds; (**a**) C-Cl and (**b**) C-H bonds of a bound DCM; (**c**–**e**) are the vinylic C-H bonds of M1; and (**f**) is the phenyl ring of triphenylphosphine (PPh_3_).

The halogen bonding is well known where the halo atom (from DCM or chloroform) behaves as electron acceptor and interacts with the electron rich species [31,32,33,34]. In the present paper, the H-bonding was observed with chlorine and it was supported by Raman spectroscopy. A RAMAN spectroscopy experiment was studied to understand the mechanism for sensitive and selective sensing of DCM towards M1. The interaction of DCM with M1 is shown in the schematic representative structural model with labeled characteristic bonds to explain the observed Raman peak (Figure 7).

**Figure 8:**
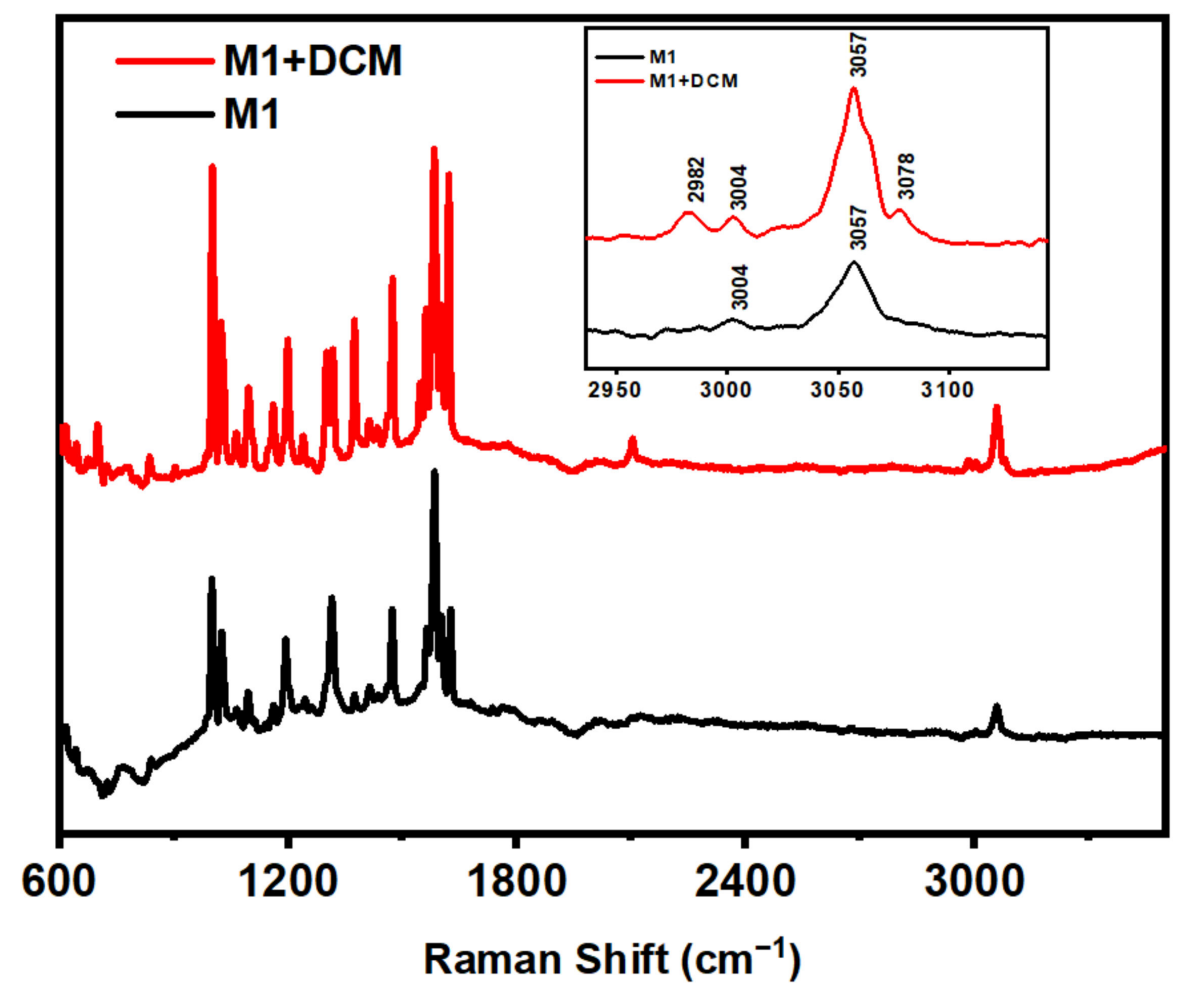
Raman spectra for solid M1 before and after DCM treatment (inset: enlarged view of several peaks).

**Figure 9:**
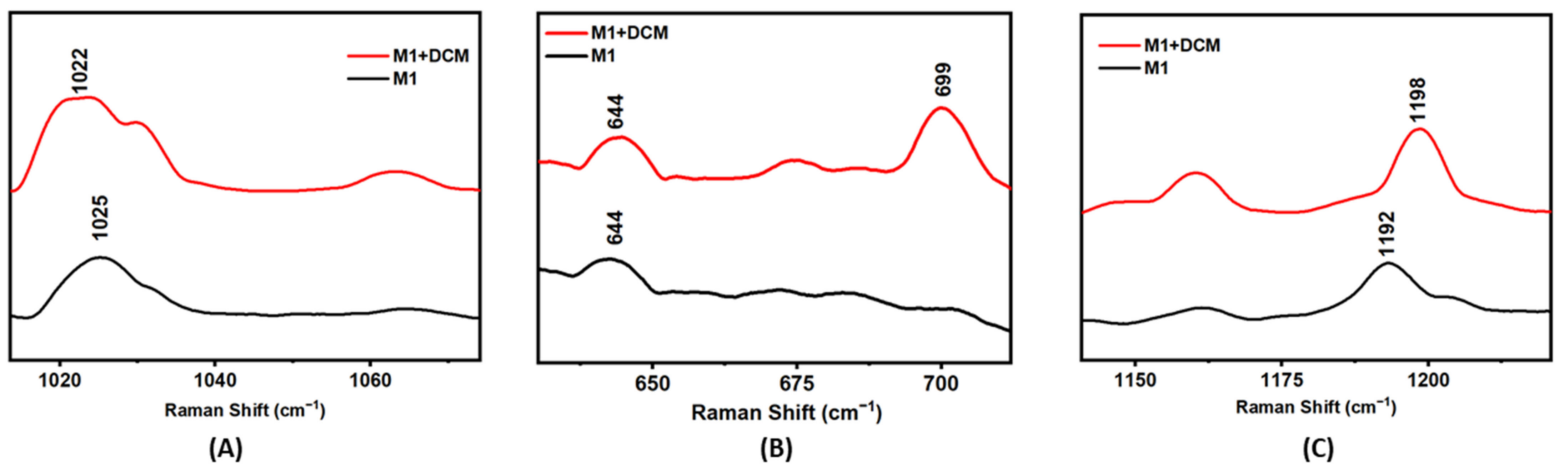
Enlarged views of RAMAN spectra (partwise): (**A**) The bending peak for C-H(c) of vinyl (=CH_2_), (**B**) the cis C-H(d) bending vibrations of vinyl (=CH_2_) and symmetric stretching C-Cl(a) of treated DCM at 699 cm^−1^, (**C**) phenyl ring vibrations; (f) for mono-substituted phenyl of triphenylphosphine (PPh_3_) increased from 1192 to 1198 cm^−1^.

The RAMAN peaks for C-Cl and C-H symmetric stretching for a bare DCM molecule were reported at 713 and 2996 cm^−1^, respectively [35]. While treating M1 with DCM, some changes in original C-Cl and C-H stretching peaks were observed. The symmetric stretching of C-Cl (a) is reduced to 699 cm^−1^ and symmetric stretching of C-H (b) is lowered from 2996 cm^−1^ to 2982 cm^−1^ (Figure 8 and Figure 9B and Table 1). The original Raman peak for the case of C-H (c) in vinylic substituent of M1 in the complex is also changed (after treatment with DCM). The bending peak for C-H (c) of substituted vinylic carbon (=CH_2_), which is trans to ligand L1 of M1 (Figure 7) originally observed at 1025 cm^−1^ for M1, is reduced to 1022 cm^−1^ (Figure 9A), with a broader and clearer observation of splitting (as compared to bare M1) (considered as monoalkyl vinyl) [36]. The other C-H stretching of vinylic parts, such as cis C-H (d), bending vibrations of vinylic carbon (=CH_2_) and a stretching C-H (e) of vinyl carbon (-CH=) observed at 644 cm^−1^ and 3057 cm^−1^, respectively, [29] and these original vibrational Raman peaks for these bonds remain unchanged as compared with the DCM treated M1 (Figure 8 and Figure 9B and Table 1).

Moreover, the asymmetrical stretching peak corresponding to C-H (c, d) of vinylic carbon (=CH_2_) of M1 is prominently evolved at 3078 cm^−1^ for the case of DCM treated M1 (the same peak was not observed for the bare M1) (Figure 8). However, the original symmetrical stretching peak for C-H (c, d) of vinylic carbon (=CH_2_) of M1 observed at 3004 cm^−1^ becomes stronger, relative to the M1 treated with DCM (Figure 8). We have carried out the Raman analysis for the M1 with dibromomethane (DBM) (Figures S13 and S14, and Table S1 †ESI) and diiodomethane (DIM) (Figures S15 and S16, and Table S2 †ESI). A change in Raman shift for M1 with DBM (similar to DCM) was observed, while no change in Raman shift was observed with DIM. After studying the Raman peaks, all the changes are centered on C-H (c) of vinylic substituent of M1 and the C-Cl of DCM. This signifies that the C-Cl (a) of DCM loosely interacts with the trans C-H (c) of vinyl (=CH_2_) of M1, while exposing to DCM and supporting the proposed model.

Furthermore, phenyl ring vibrations (f) for a phenyl of triphenylphosphine (PPh_3_) increased from 1192 to 1198 cm^−1^ (Figure 7 and Figure 9C and Table 1) [37]. The fact can be rationalized with the lowering of the vibrations of the phenyl ring because of the restriction imposed by the DCM (Figure 7 and Figure 9C). As a result, the turn-on emission was observed [25,38]. This turn-on emission further supported a low-temperature experiment (Figure S17, †ESI). The non-emissive powdered M1 shows the same yellow emission at low temperature where turn-on luminescence will be observed by restricted intramolecular motion [39]. On studying the FESEM image, M1 shows an inter-linked nano-rod and non-porous microstructure. An FESEM image of DCM treated M1 (after treating with DCM) results in a totally different morphology, i.e., a porous with a honey-comb type of structure (Figure S18, †ESI). The restriction on the movement of some part of M1 (vinyl and PPh_3_ units) results from the DCM molecule’s interactive force, which may lead to the observed microporous nature.

Thermogravimetric analysis (TGA) gives an understanding of the thermal stability of a sample by giving a weight loss for M1, and DCM treated M1 (Figure S19, †ESI). The synthesized M1 is thermally stable up to 250 °C. The sample holder containing powdered M1 was exposed to DCM and kept in open air for 5 min to evaporate excess DCM. The DCM treated M1 analyzed by TGA was observed to have around 13% weight loss at up to 80 °C. It supports the presence of DCM, which is loosely bound to M1.

## 3. Materials and Methods

### 3.1. Materials

The starting materials 4-vinylphenylboronic acid, tetrakis(triphenylphosphine)palladium (0), iridium(III) chloride hydrate were purchased from TCI chemicals (Tokyo, Japan). 2-bromopyridine was purchased from Alfa Aesar company (Thermo Fisher Scientific, Kandel, Germany). Potassium carbonate (K_2_CO_3_), Sodium carbonate (Na_2_CO_3_), triphenylphosphine (PPh_3_), 2-ethoxyethanol, and the UV grade solvents (tetrahydrofuran (THF), hexane, toluene, ethanol, and polyethylene glycol (PEG) were procured from Spectrochem (Mumbai, India), Merck company (Darmstadt, Germany).

### 3.2. Instrumentation

^1^H-NMR, ^13^C-NMR and ^31^P-NMR spectra were recorded using a 400 MHz Brucker NMR spectroscope (Billerica, MA, USA). High-resolution mass spectra were recorded on Agilent 6545 Q-TOF LC/MS. UV-VIS absorption spectra were recorded using a Shimadzu Spectrophotometer (model UV-1800 and 2550, Kyoto, Japan). The Steady-state photoluminescence (PL) spectra were recorded on a Horiba Jobin Yvon Spectrofluorometer (FluoroMax-4) and Horiba ‘FluoroLog-3′ Spectrofluorimeter (Tokyo, Japan). The variation in particle size in aggregated state was recorded by dynamic light scattering (DLS) experiment, using Anton Paar Litesizer 500 at 25 °C temperature. Raman analysis studied by HORIBASCI Raman instrument (model no. LabRAM HR EVO) using excitation lasers with 633 nm wavelength. Microscope version, XT Platform version, XT UI version, Modal- ‘‘APREO S’’ FE-SEM was used to investigate the morphology of the synthesized M1 sample. Thermogravimetric analysis (TGA) was performed by TGA-50, SHIMADZU equipment at 10 °C/min, under a nitrogen atmosphere.

### 3.3. Experimental Procedure

#### 3.3.1. Synthesis of Ligand L1

In a 250 mL round bottom flask, a mixture of 4-vinylphenylboronic acid (1.31 g, 8.85 mmol), K_2_CO_3_ (3.29 g, 10.12 mmol), toluene: ethanol (10:1, 22 mL), 2-bromopyridine (1 g, 6.32 mmol) stirred under N_2_ gas for 5 min. The catalyst tetrakis (triphenylphosphine)palladium(0) added and refluxed for 5 h at 100 °C temperature. Then, it was cooled to room temperature. The reaction was quenched with the addition of water and washed with brine solution, then extracted with ethyl acetate finally dried with anhydrous Na_2_SO_4,_ (filtration over celite to remove insoluble palladium catalyst residues). The crude product was purified by column chromatography, with Hexane: Ethyl acetate (95:5) as eluent. A colorless oil isolated as the desired vinyl substituted phenylpyridine ligand (yield, 83.8%).

^1^H-NMR (400 MHz, Chloroform-*d*) δ 8.72 (d, *J* = 4.6 Hz, 1H), 8.00 (d, *J* = 8.4 Hz, 2H), 7.81–7.72 (m, 2H), 7.55 (d, *J* = 8.3 Hz, 2H), 7.25 (ddd, *J* = 6.0, 4.9, 2.6 Hz, 1H), 6.80 (dd, *J* = 17.6, 10.9 Hz, 1H), 5.85 (dd, *J* = 17.6, 0.9 Hz, 1H), 5.33 (dd, *J* = 10.9, 0.9 Hz, 1H) (Figure S1).^13^C-NMR (101 MHz, Chloroform-*d*) δ 157.01, 149.68, 138.70, 138.20, 136.74, 136.40, 127.04, 126.62, 122.09, 120.42, 114.49 (Figure S2).

#### 3.3.2. Synthesis of Iridium Complex M1

In a 50 mL round bottom flask, a mixture of iridium(III) chloride hydrate (0.216 g, 1.19 mmol), triphenylphosphine (0.650 g, 2047 mmol) and 4 mL of 2-ethoxyethanol solvent, stirred for 3 h at 125 °C temperature. While stirring the mixture for 3 h, it turned into yellow colored liquid. Then, L1 (0.216 g, 1.19 mmol) and Na_2_CO_3_ (0.432 g, 4.07 mmol) added and refluxed for 3 h at 125 °C temperature under the nitrogen gas. The reaction was quenched with the addition of water, then extracted with ethyl acetate finally dried with anhydrous Na_2_SO_4_. The crude product was purified by column chromatography, with Hexane: Ethyl acetate (80:20) as eluent. Yellow colored solid isolated as desired M1 with 40% yield. The product was characterized by ^1^H, ^13^C and ^31^P-NMR spectra.

^1^H-NMR (400 MHz, Chloroform-*d*) δ 8.97 (d, *J* = 5.6 Hz, 1H), 7.42–7.36 (m, 15H), 7.18–7.15 (m, 7H), 7.12 (dt, *J* = 7.4, 1.2 Hz, 11H), 6.65 (dt, *J* = 7.1, 1.6 Hz, 2H), 6.29 (s, 1H), 5.95 (dd, *J* = 17.5, 10.8 Hz, 1H), 5.13 (dd, *J* = 17.5, 1.4 Hz, 1H), 4.85 (dd, *J* = 10.8, 1.4 Hz, 1H), −16.82 (s, 1H) (Figure S3).

^13^C-NMR (101 MHz, Chloroform-*d*) δ 149.78, 141.77, 137.61, 135.29, 134.14, 134.07, 134.02, 133.96, 131.94, 131.67, 131.41, 129.10, 128.99, 127.21, 127.16, 127.12, 122.30, 120.35, 117.61, 117.19, 112.30 (Figure 4).

^31^P-NMR (162 MHz, Chloroform-*d*) δ 9.01 (Figure S5).

ESI-HRMS calculated: [M-Cl]^+^: *m*/*z* = 898.21; found: [M-Cl]^+^: *m*/*z* = 898.23 (Figure S6).

## 4. Conclusions

In this work, a new AIE active mono-cyclometalated Ir(III) complex M1 synthesized by a simple and straightforward reaction pathway which shows vapochromic dichloromethane (DCM) detection. The M1 exhibits a bright turn-on emission in the presence of DCM vapor (λ_emi_, 530 nm). The mechanism of DCM sensing was explored by Raman spectroscopy. The notable achievements are the selective and sensitive detection of DCM and the reusability of probe M1. The observed DCM detection limit is 4.9 ppm in the vapor-phase with pristine M1. To the best of our knowledge, the observed LOD for the detection of DCM vapor with pristine probe showed the best result. Moreover, such a technique for the detection of DCM would be cost-effective and shows potentiality for commercialization. The work should inspire the development of convenient routes for the development of new materials to detect VOCs from the workplace in industries/laboratories.

## Figures and Tables

**Table 1:** Various vibrational modes for M1 and DCM treated M1.

	Raman Shifts (cm^−1^)			
Vibrational Modes	DCM only	DCM (in M1+DCM)	M1 only	M1 (in M1+DCM)
C-Cl symmetric stretching (a)	713	699	-	-
C-H symmetric stretching (b)	2996	2982	-	-
C-H bending (c)	-	-	1025	1022
C-H bending (d)	-	-	644	644
C-H stretching (e)	-	-	3057	3057
C-H antisymmetric stretching of vinylic carbon (=CH_2_) (c, d)	-	-	-	3078
C-H symmetric stretching of vinylic carbon (=CH_2_) (c, d)	-	-	3004	3004
Phenyl ring vibrations of triphenylphosphine (PPh_3_) (f)	-	-	1192	1198

(a–f) are the notations for the labeled characteristic bonds from Figure 7.

## Data Availability

Data supporting the findings of this study are available within the article and Supplementary Materials.

## Supplementary Materials

The following are available online at. Figures S1 and S2: ^1^H, ^13^C-NMR spectrum of L1; Figures S3–S5: ^1^H, ^13^C, ^31^P-NMR spectrums of M1; Figure S6: Mass spectrum of compound M1; Figure S7: DLS particle size distribution plot of M1; Figure S8: AIE study of M1 in tetrahydrofuran (THF): polyethylene glycol (PEG) mixtures; Figure S9: The optimized geometry of M1; Figure S10: Emission plot for powdered M1 with DCM and DBM; Figure S11: Photograph of DCM vapor sensing setup; Figure S12: Calculation of limit of detection for M1 in vapor phase; Figures S13 and S14: Schematic representation for the structural model of interaction of DBM with M1 and respective Raman plot; Table S1: Various vibrational modes for M1 and DBM treated M1; Figure S15 and S16: Schematic representative structural model of interaction of DIM with M1 and respective Raman plot; Table S2: Various vibrational modes for M1 and DBM treated M1; Figure S17: (a) Photograph of low temperature study for M1; Figure S18: FESEM images for before and after addition of DCM to powdered M1; Figure S19: TGA plot of M1 for before and after DCM treated.

## Sample Availability

Samples of the compounds are not available from the authors.
