# Supplementary material for: ‘Aggregation-Induced Emission’ Active Mono-Cyclometalated Iridium(III) Complex Mediated Efficient Vapor-Phase Detection of Dichloromethane
Source: Molecules. 2021 Dec 29;27(1):202. doi: 10.3390/molecules27010202 (PMC8746431; doi:10.3390/molecules27010202)
Supplement: Supplementary file 1 [file molecules-27-00202-s001.zip › molecules-1484911-supplementary.pdf]

molecules

MDPI

Supplementary Material

'Aggregation-Induced Emission' Active Mono-Cyclometalated Iridium(III) Complex Mediated Efficient Vapor-Phase Detection of Dichloromethane

Pramod C. Raichure [1], Vishal Kachwal [1,2] and Inamur Rahaman Laskar [1,*]

[1] Department of Chemistry, Birla Institute of Technology and Science, Pilani Campus, Pilani 333031, Rajasthan, India; raichurepramod@gmail.com (P.C.R.); vishkachwal4@gmail.com (V.K.)
[2] Department of Engineering Science, University of Oxford, Oxford OX1 3PJ, UK
* Correspondence: ir_laskar@pilani.bits-pilani.ac.in

Contents

Figure S1. ^{1}H NMR spectrum of L1 in $CDCl_3$ solvent.

Figure S2. ^{13}C NMR spectrum of L1 in $CDCl_3$ solvent.

Figure S3. ^{1}H NMR spectrum of M1 in $CDCl_3$ solvent.

Figure S4. ^{13}C NMR spectrum of M1 in $CDCl_3$ solvent.

Figure S5. ^{31}P NMR spectrum of M1 in $CDCl_3$ solvent.

Figure S6. Mass spectrum of compound M1

Figure S7. DLS particle size distribution plot of particle (size) diameter in nm *vs* distribution (percent) of solution of M1 in THF for 0% hexane (green coloured) and for 90% hexane (orange coloured) in THF: hexane mixture. The particle size for 0% hexane is 52.3 nm with PDI 0.2 (26.4%) and for 90% hexane is 170.5 nm with PDI 1.1 (115.4%).

Figure S8. (a) Photograph of M1 in tetrahydrofuran (THF): polyethylene glycol (PEG) mixtures with different PEG fractions upon UV light irradiation (at λ_{ex}= 365 nm). (b) Photoluminescence (PL) spectra for the same mixture under excitation 360 nm wavelength. (c) Line plot for the changes in PL intensity for the M1 with different PEG fractions.

Figure S9. The optimized geometry; HOMO and LUMO molecular orbitals of M1 by DFT-based calculation *via* Gaussian 09 with the LanL2DZ basis set; (a) basic unit of M1, (b) molecular orbitals structure of M1, (c) LUMO, (d) HOMO.

Figure S10. (a) Photographs of emission of powdered M1 with DCM and DBM on glass film under the UV lamp (365 nm), (b) PL spectra for the same under 360 nm excitation.

Figure S11. Photograph of DCM vapor sensing setup; enlarged view of DCM container connected with glass separating funnel (25 ml volume).

Figure S12. Plot of DCM vapor (kPa) *vs* PL intensity (Pearson's r value= 0.99) and calculation of limit of detection for M1 in vapor phase with conversion of vapor pressure from kPa to ppm.

Figure S13. (A) Schematic representation for the structural model of interaction of DBM with M1 labeling with characteristic bonds: C-Br (a) and C-H (b) bonds of a bound DBM; (c), (d) and (e) are the vinylic C-H bonds of M1; and (f) is the phenyl ring of triphenylphosphine (PPh_3), (B) Raman spectra for solid M1 before and after DBM treatment (inset: enlarged view of several peaks).

Figure S14. Enlarged views of RAMAN spectra for DBM with M1 (partwise): (A) The bending peak for C-H(c) of vinyl (=CH_2), (B) phenyl ring vibrations(f) (for mono-substituted phenyl of triphenylphosphine (PPh_3) increased from 1192 to 1199 cm^{-1}).

Table S1. Various vibrational modes for M1 and DBM treated M1.

Figure S15. (A) Schematic representative structural model of interaction of DIM with M1 labeling with characteristic bonds; (a) C-I and (b) C-H bonds of a bound DIM; (c), (d)

and (e) are the vinylic C-H bonds of M1; and (f) is the phenyl ring of triphenylphosphine (PPh_3), (B) Raman spectra for solid M1 before and after DIM treatment (inset: enlarged view of several peaks).

Figure S16. Enlarged views of RAMAN spectra for DIM with M1 (partwise): (A) The bending peak for C-H(c) of vinyl (=CH_2), (B) phenyl ring vibrations(f); for mono-substituted phenyl of triphenylphosphine (PPh_3) 1192 cm^{-1}.

Table S2. Various vibrational modes for M1 and DBM treated M1.

Figure S17. (a) Photograph of a glass vial containing little amount of non-emissive powdered M1 at room temperature under the UV lamp (365 nm); same glass vial deepens into the liquid nitrogen for 10 sec. to achieve low temperature, (b) Photograph of same glass vial under the UV lamp (365 nm) immediately after taking out from liquid nitrogen and it observed same emission.

Figure S18. FESEM images for (a) before and (b) after addition of DCM to powdered M1.

Figure S19. TGA plot, (a) for compound M1; (b) for DCM treated M1.

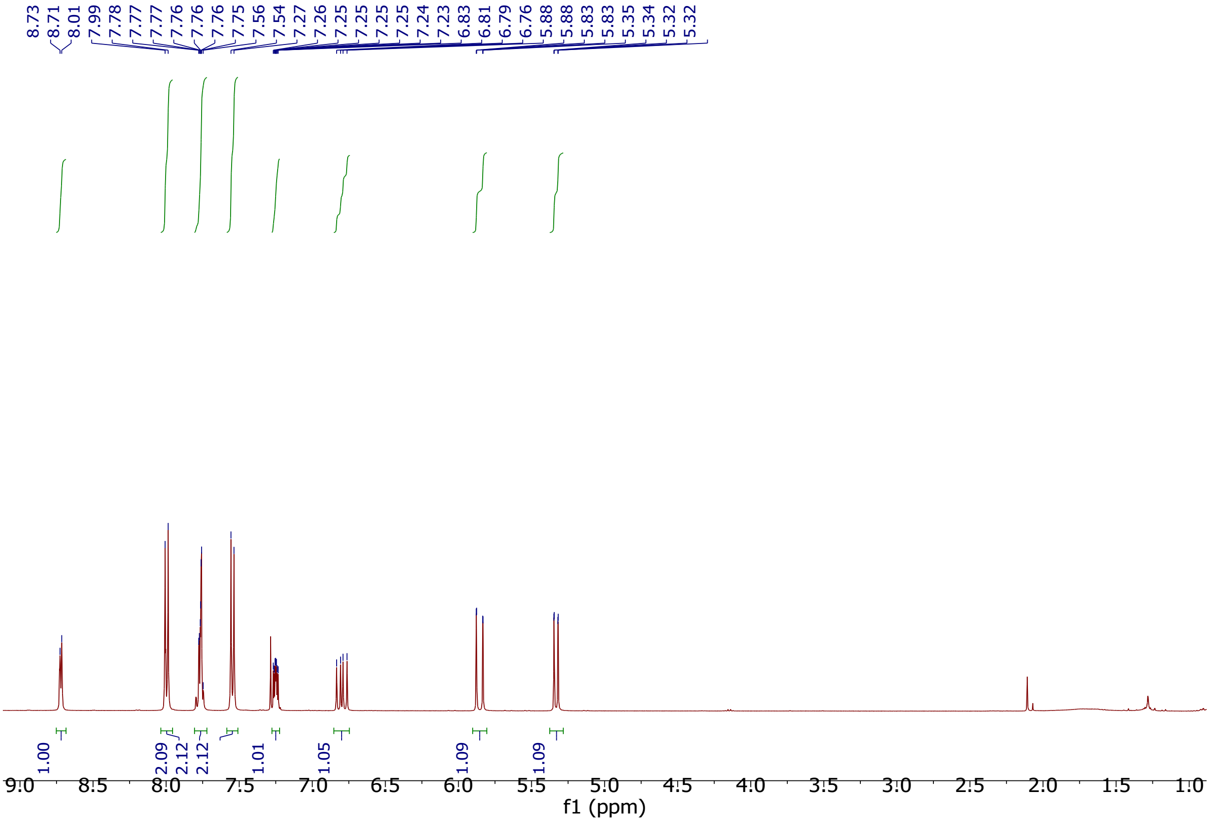

Figure S1. ^1H NMR spectrum of L1 in $CDCl_3$ solvent.

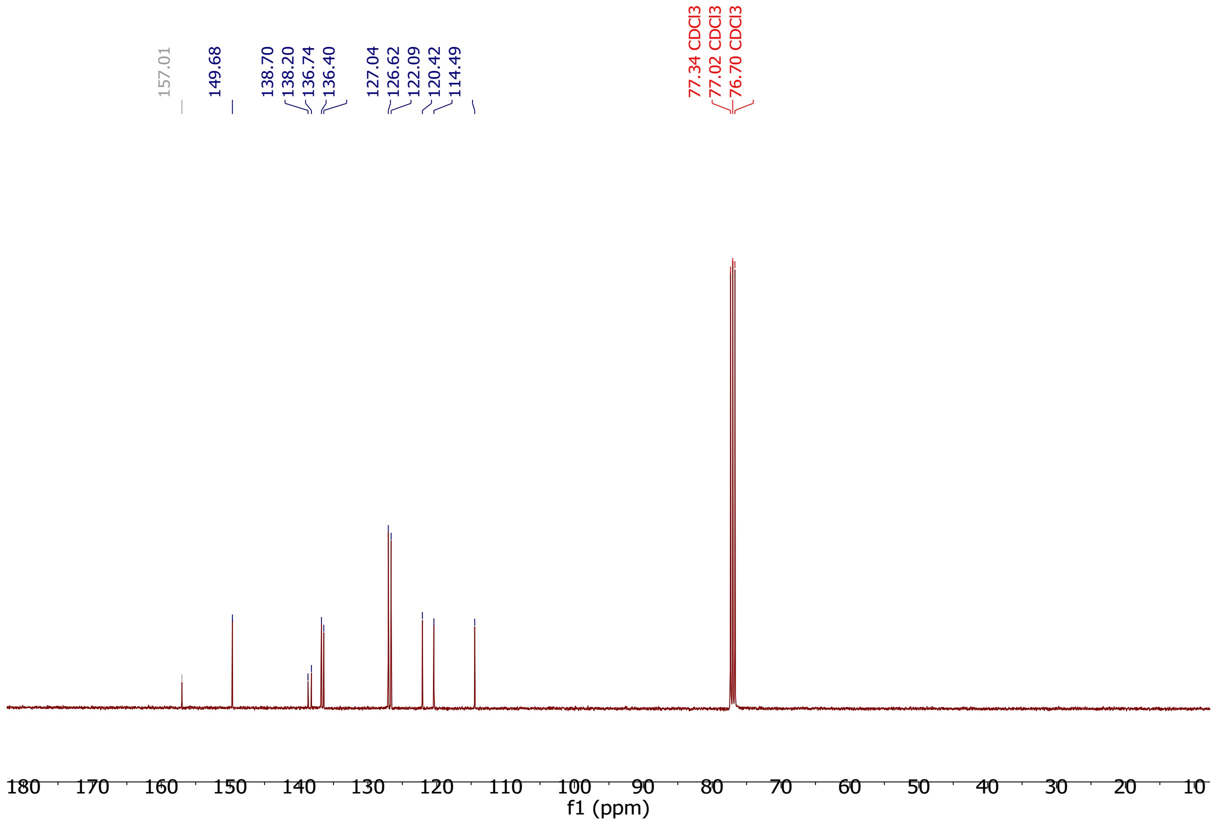

Figure S2. ^{13}C NMR spectrum of L1 in $CDCl_3$ solvent.

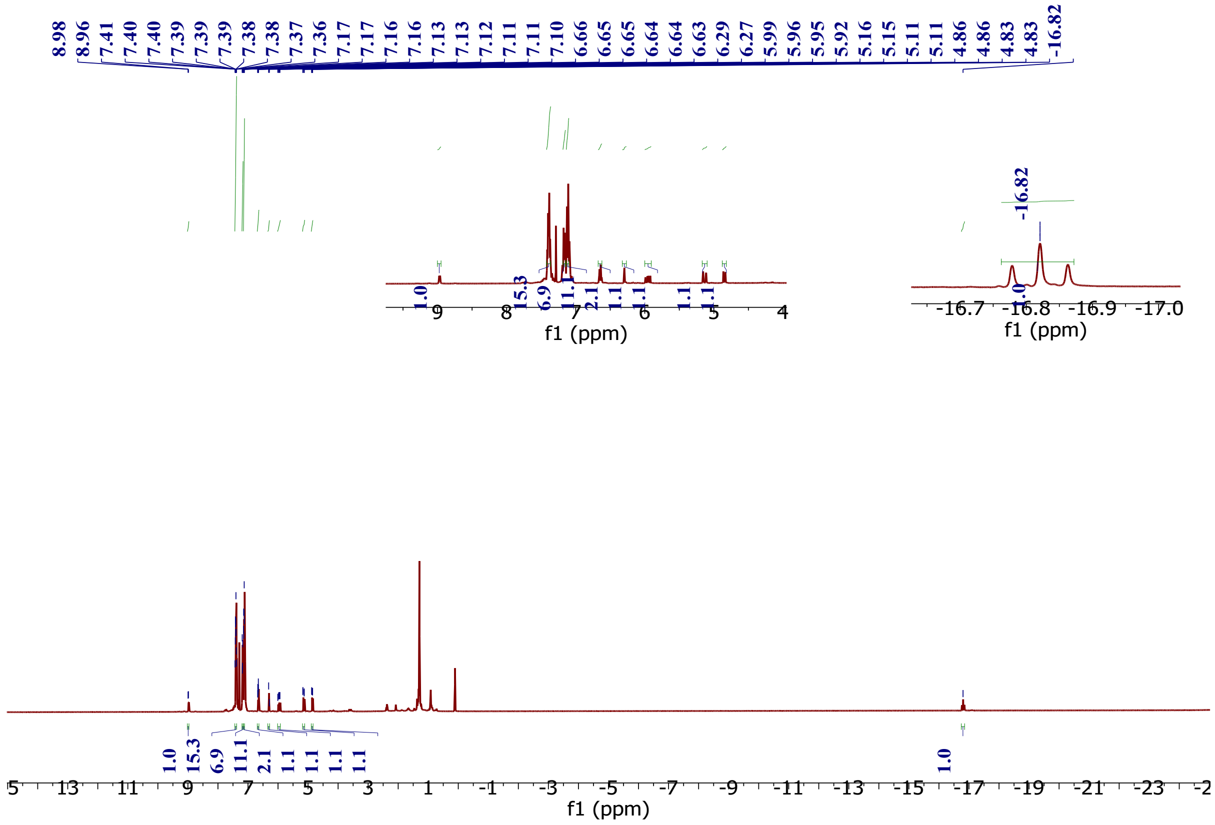

Figure S3. ^{1}H NMR spectrum of M1 in $CDCl_3$ solvent.

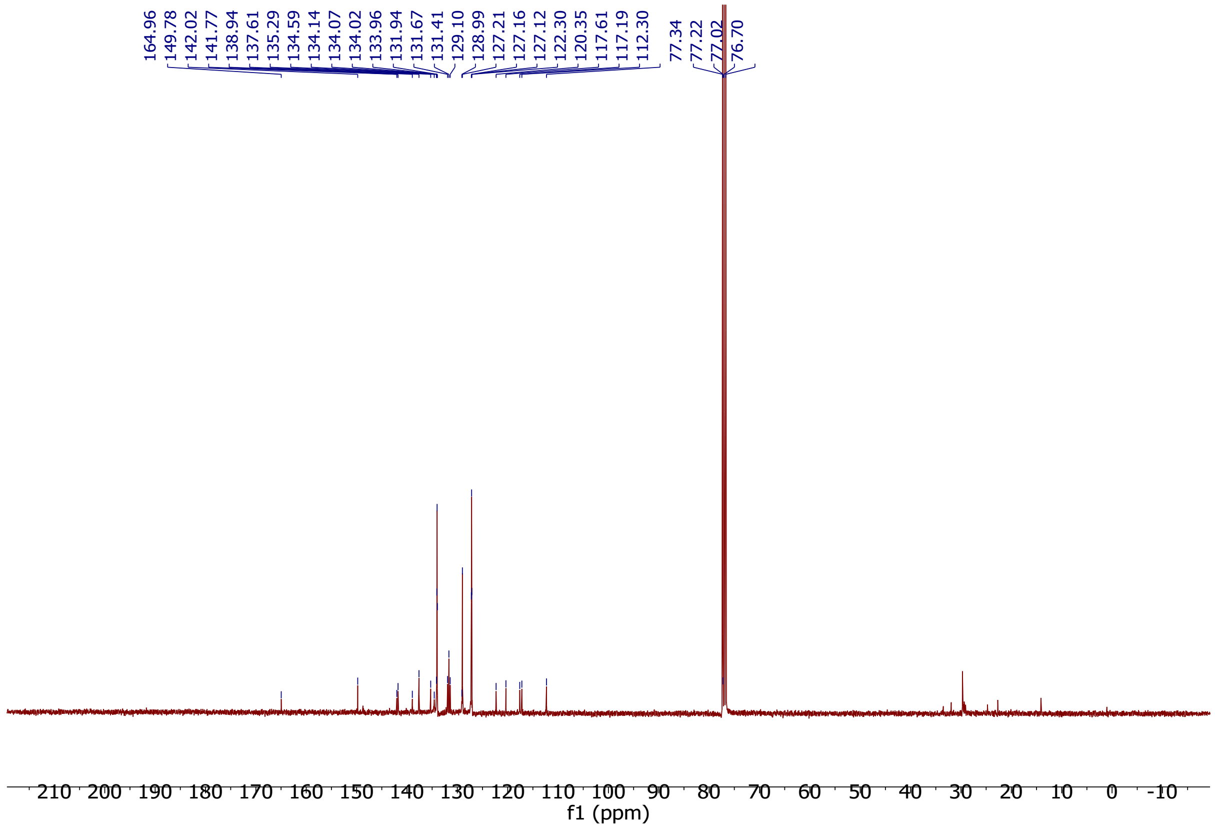

Figure S4. ^{13}C NMR spectrum of M1 in $CDCl_3$ solvent.

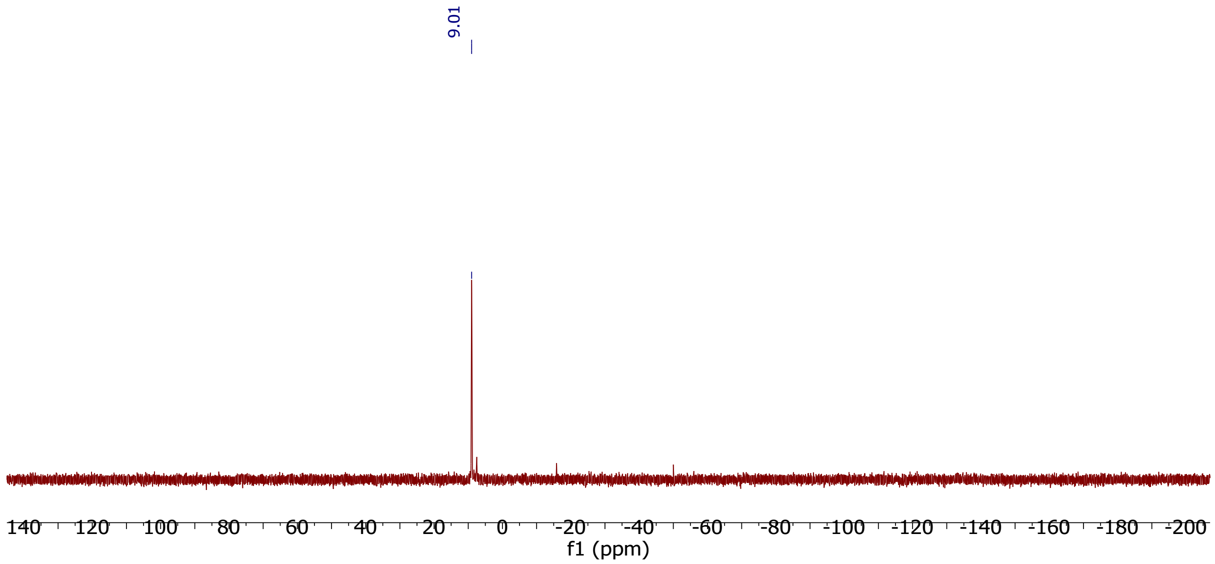

Figure S5. ^{31}P NMR spectrum of M1 in $CDCl_3$ solvent.

MS zoomed spectra:

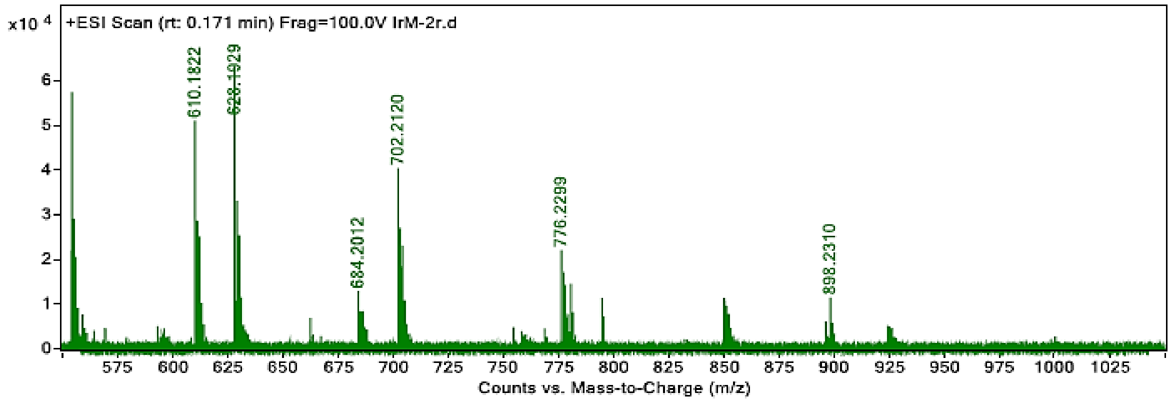

Figure S6. Mass spectrum of compound M1.

Name	Color	Hydrodyn. diam. [nm]	Polydispersity index [%]
00%	green	52.31	26.4
90%	orange	170.54	115.4

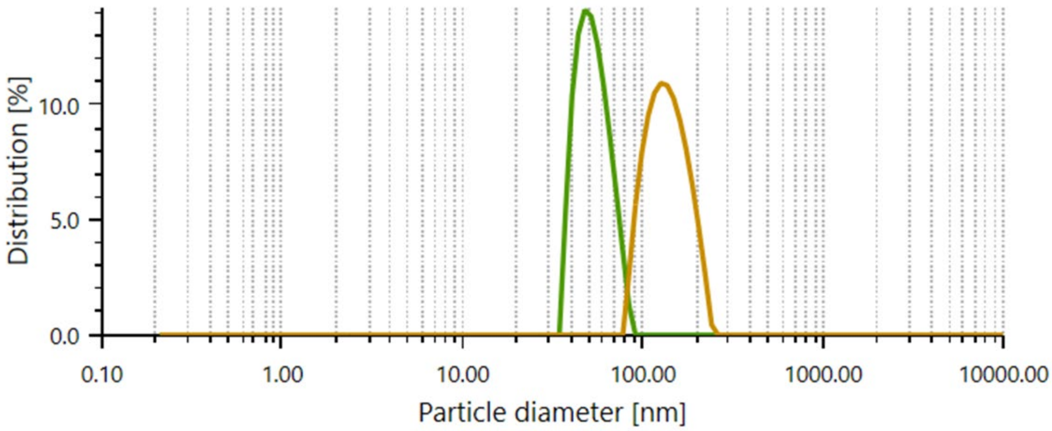

Figure S7. DLS particle size distribution plot of particle (size) diameter in nm *vs* distribution (percent) of solution of M1 in THF for 0% hexane (green coloured) and for 90% hexane (orange coloured) in THF: hexane mixture. The average particle size for 0% hexane is 52.3 nm with PDI 0.2 (26.4%) and for 90% hexane is 170.5 nm with PDI 1.1 (115.4%).

AIE EXPERIMENT: The AIE experiment for M1 carried out by taking a mixture of THF and hexane solvents. Firstly, the probe solution (10^{-3}M) for M1 was prepared in THF. The 0.5 ml of probe solution added in four glass vials of 5 ml volume, and then equally filled with THF: hexane (or PEG) mixture by changing the percentage of hexane fractions. The photoluminescence (PL) spectra of all the prepared solutions were recorded and it indicates the PL intensity steadily increases with increasing hexane fractions. To proof the RIR effect, the series of solutions of probe of THF and PEG mixture were prepared in the similar manner as descriebd above (PEG was added in place of hexane).

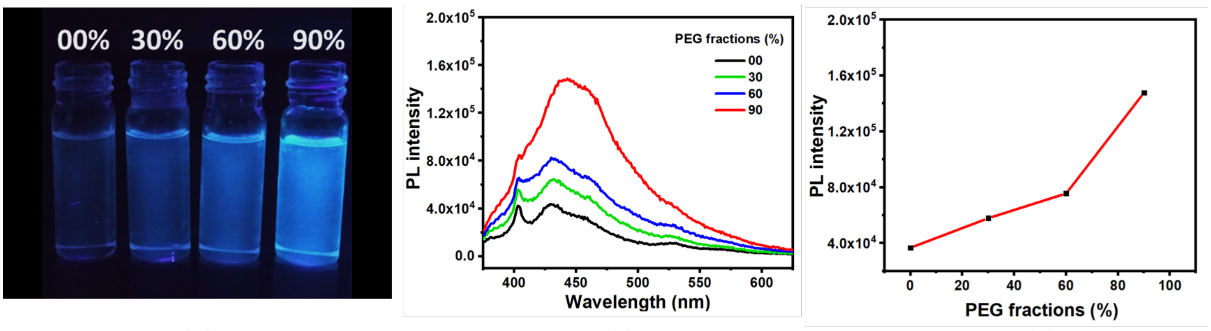

(a) (b) (c)

Figure S8. (**a**) Photograph of M1 in tetrahydrofuran (THF): polyethylene glycol (PEG) mixtures with different PEG fractions upon UV light irradiation (at λ_{ex}= 365 nm), (**b**) Photoluminescence (PL) spectra for the same mixture under excitation 360 nm wavelength. (**c**) Line plot for the changes in PL intensity for the M1 with different PEG fractions.

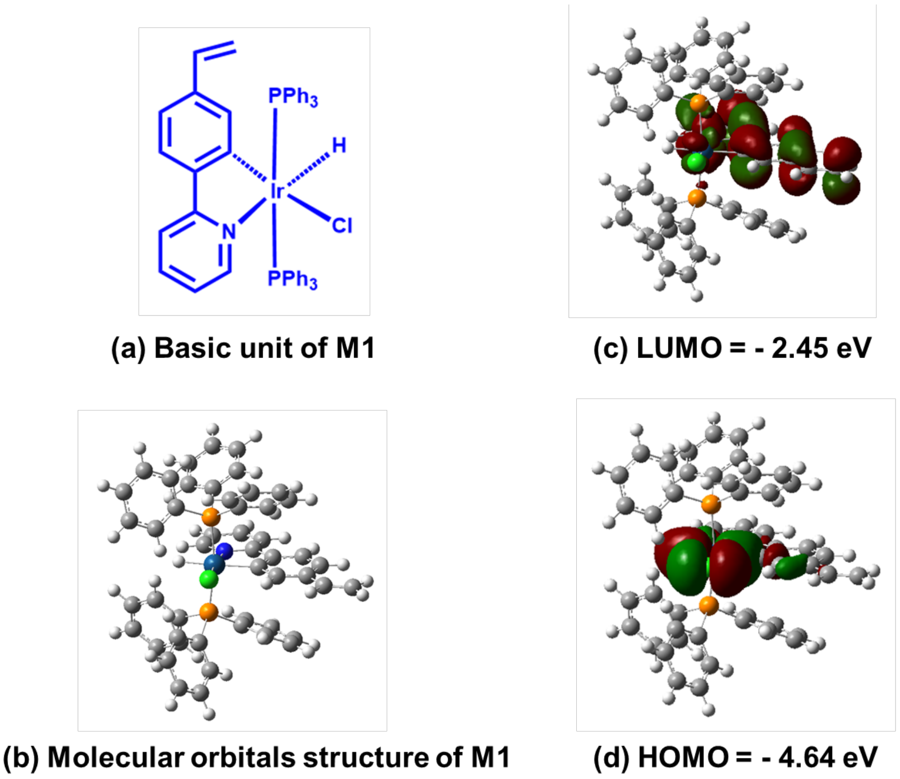

Figure S9. The optimized geometry; HOMO and LUMO molecular orbitals of M1 by DFT-based calculation *via* Gaussian 09 with the LanL2DZ basis set; (**a**) basic unit of M1, (**b**) molecular orbitals structure of M1, (**c**) LUMO, (**d**) HOMO.

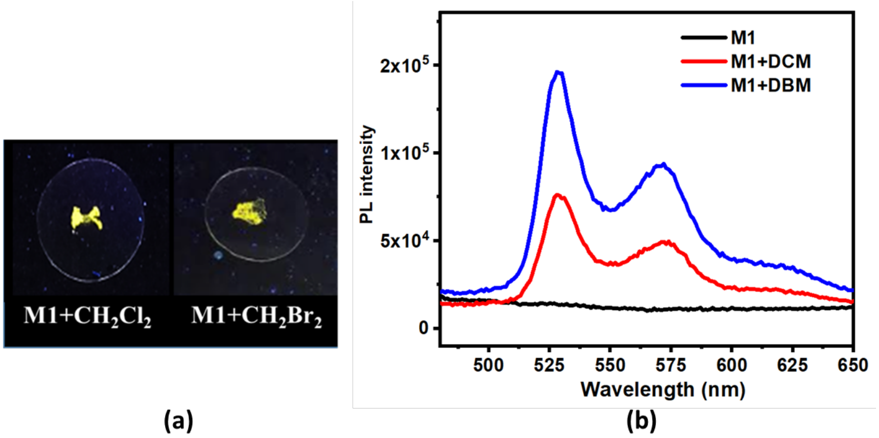

Figure S10. (**a**) Photographs of emission of powdered M1 with DCM (CH_2Cl_2) and DBM (CH_2Br_2) on glass film under the UV lamp (365 nm), (**b**) PL spectra for the same under 360 nm excitation.

DCM VAPOR DETECTION EXPERIMENT: The DCM container closed and kept in oil bath and set 33°C constant temperature to generate saturated vapor pressure. The sample holder containing powdered M1 exposed to DCM vapor for 30 sec. at outlet of glass funnel for each reading.

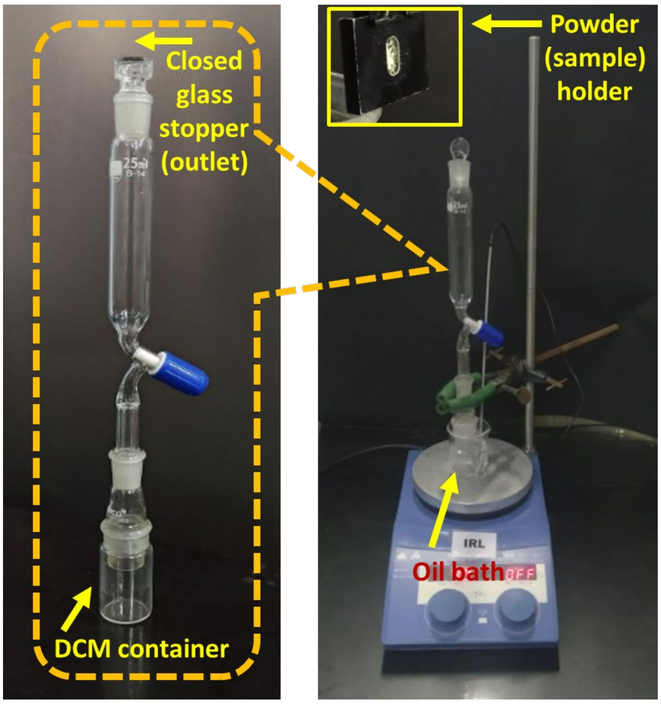

Figure S11. Photograph of DCM vapor sensing setup; enlarged view of DCM container connected with glass separating funnel (25 ml volume).

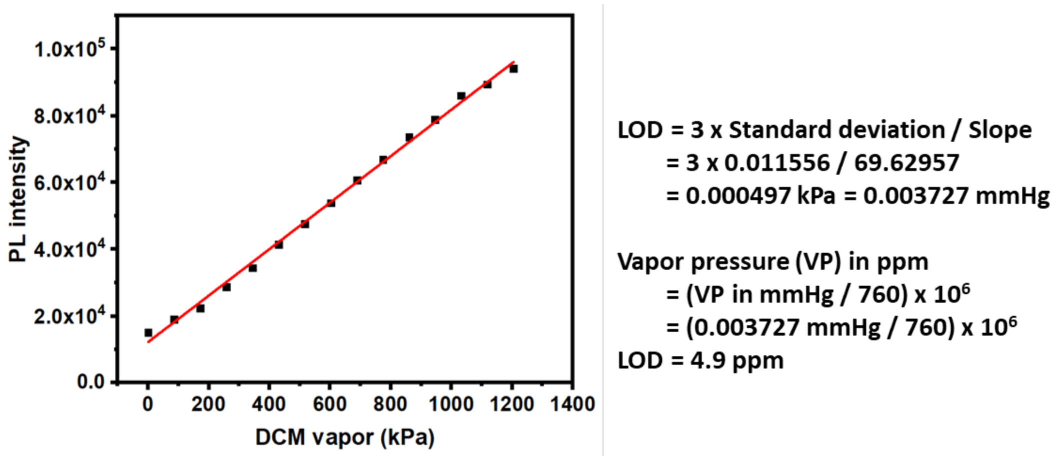

Figure S12. Plot of DCM vapor (kPa) *vs* PL intensity (Pearson's r value= 0.99) and calculation of limit of detection for M1 in vapor phase with conversion of vapor pressure from kPa to ppm.

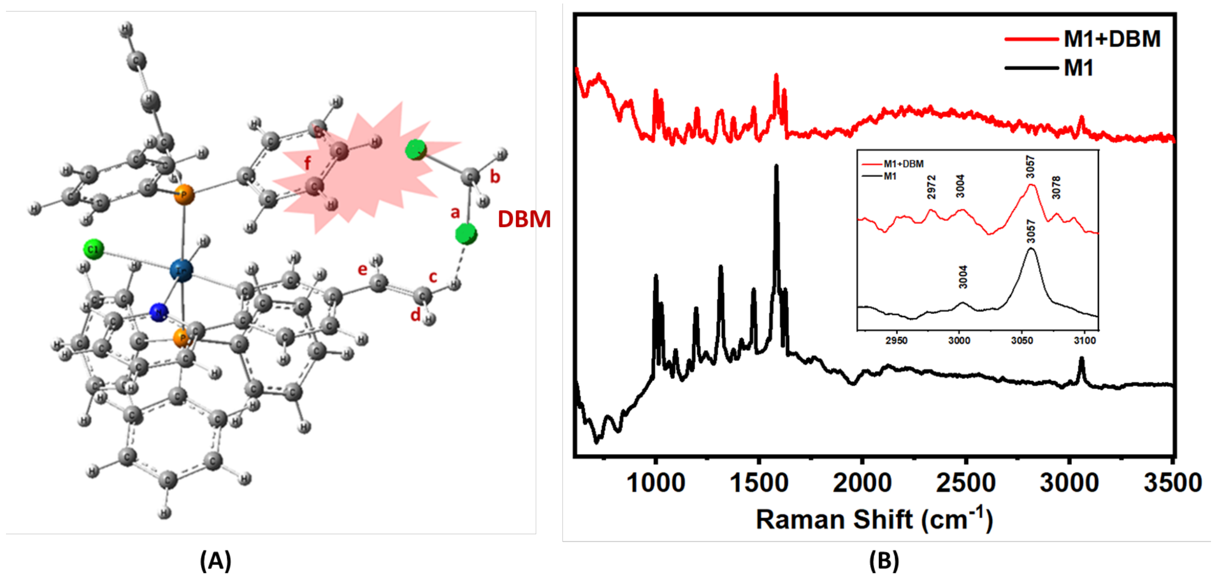

Figure S13. (**A**) Schematic representation for the structural model of interaction of DBM with M1 labeling with characteristic bonds: C-Br (a) and C-H (b) bonds of a bound DBM; (c), (d) and (e) are the vinylic C-H bonds of M1; and (f) is the phenyl ring of triphenylphosphine (PPh_3), (**B**) Raman spectra for solid M1 before and after DBM treatment (inset: enlarged view of several peaks).

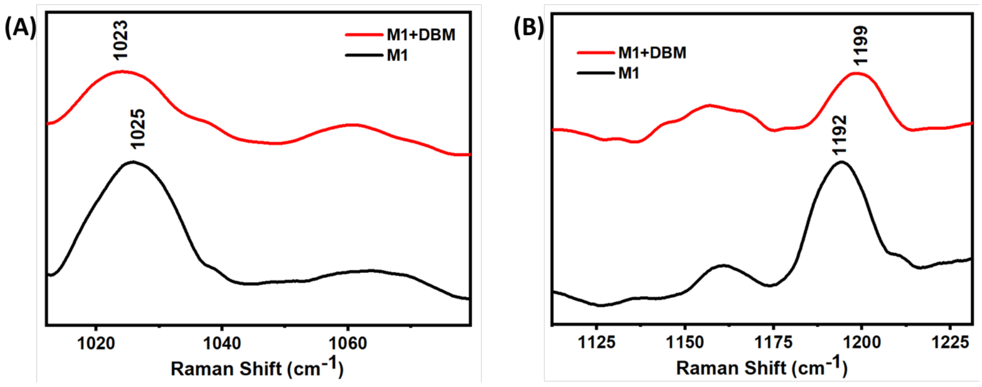

Figure S14. Enlarged views of RAMAN spectra for DBM with M1 (partwise): (**A**) The bending peak for C-H(c) of vinyl ($=CH_2$), (**B**) phenyl ring vibrations(f) (for mono-substituted phenyl of triphenylphosphine (PPh_3) increased from 1192 to 1199 cm^{-1}).

Table S1. Various vibrational modes for M1 and DBM treated M1.

	Raman Shift (cm^{-1})	
**Vibrational Modes**	**M1 only**	**M1 (in M1 + DBM)**
C-H bending (c)	1025	1023
C-H stretching (e)	3057	3057
C-H antisymmetric stretching of vinylic carbon (=CH_2) (c, d)	--	3078
C-H symmetric stretching of vinylic carbon (=CH_2) (c, d)	3004	3004
Phenyl ring vibrations of triphenylphosphine (PPh_3) (f)	1192	1199

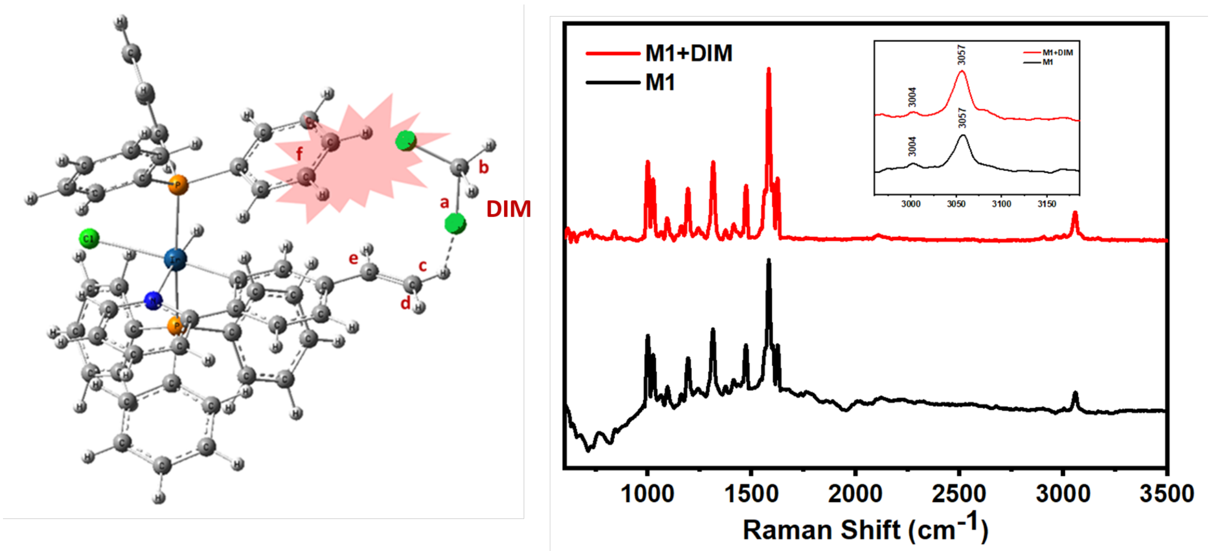

Figure S15. (**A**) Schematic representative structural model of interaction of DIM with M1 labeling with characteristic bonds; (a) C-I and (b) C-H bonds of a bound DIM; (c), (d) and (e) are the vinylic C-H bonds of M1; and (f) is the phenyl ring of triphenylphosphine (PPh_3), (**B**) Raman spectra for solid M1 before and after DIM treatment (inset: enlarged view of several peaks).

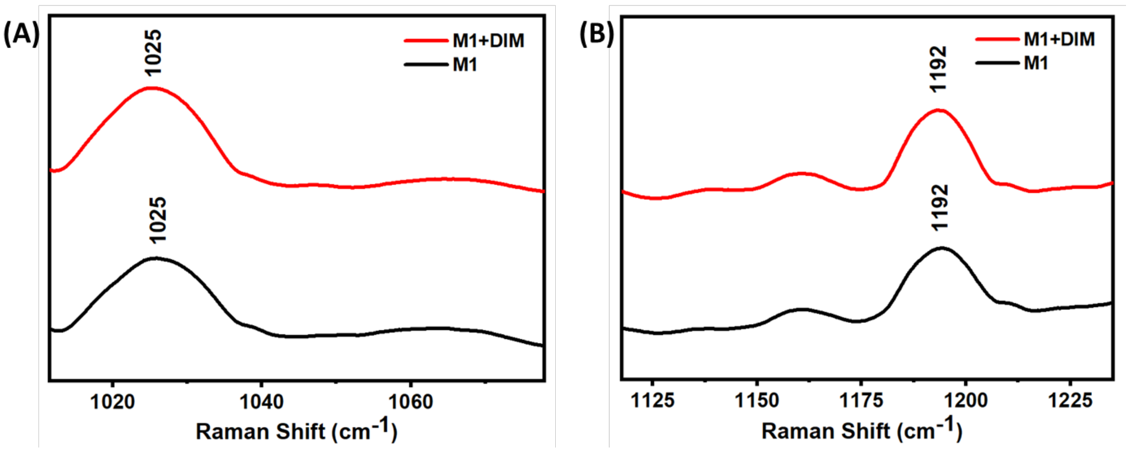

Figure S16. Enlarged views of RAMAN spectra for DIM with M1 (partwise): (**A**) The bending peak for C-H(c) of vinyl (=CH_2), (**B**) phenyl ring vibrations(f); for mono-substituted phenyl of triphenylphosphine (PPh_3) 1192 cm^{-1}.

Table S2. Various vibrational modes for M1 and DIM treated M1.

	Raman Shift (cm^{-1})	
**Vibrational modes**	**M1 only**	**M1 (in M1 + DIM)**
C-H bending (c)	1025	1025
C-H stretching (e)	3057	3057
C-H antisymmetric stretching of vinylic carbon (=CH_2) (c, d)	--	--
C-H symmetric stretching of vinylic carbon (=CH_2) (c, d)	3004	3004
Phenyl ring vibrations of triphenylphosphine (PPh_3) (f)	1192	1192

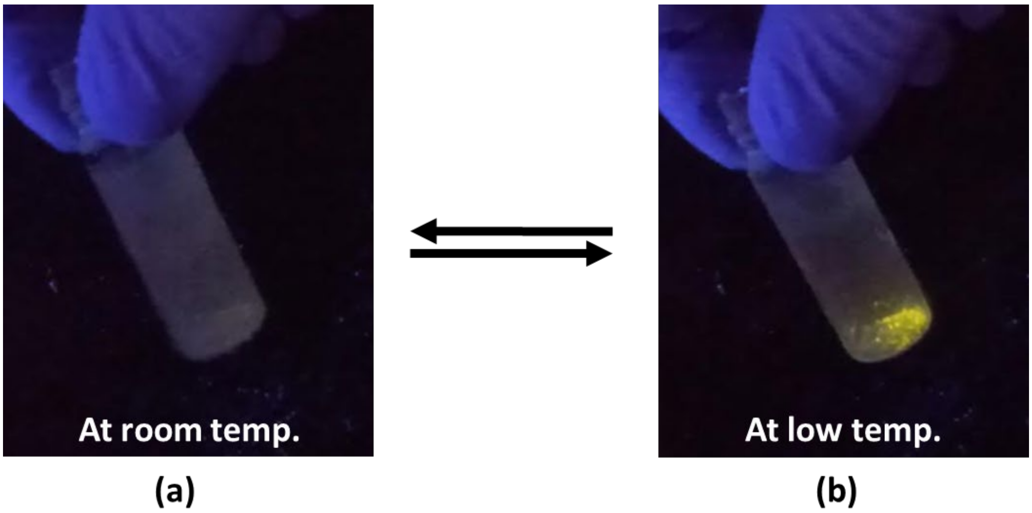

Figure S17. (**a**) Photograph of a glass vial containing little amount of non-emissive powdered M1 at room temperature under the UV lamp (365 nm); same glass vial dipped into the liquid nitrogen for 10 sec. to achieve low temperature, (**b**) Photograph of same glass vial under the UV lamp (365 nm) immediately after taking out from liquid nitrogen and it observed same emission with the one observed in presence of DCM.

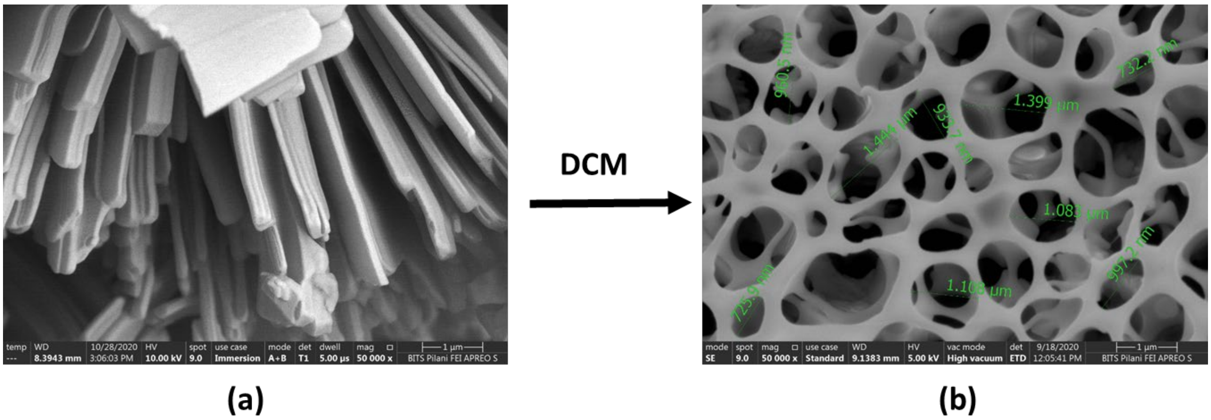

Figure S18. FESEM images for (**a**) before and (**b**) after addition of DCM to powdered M1.

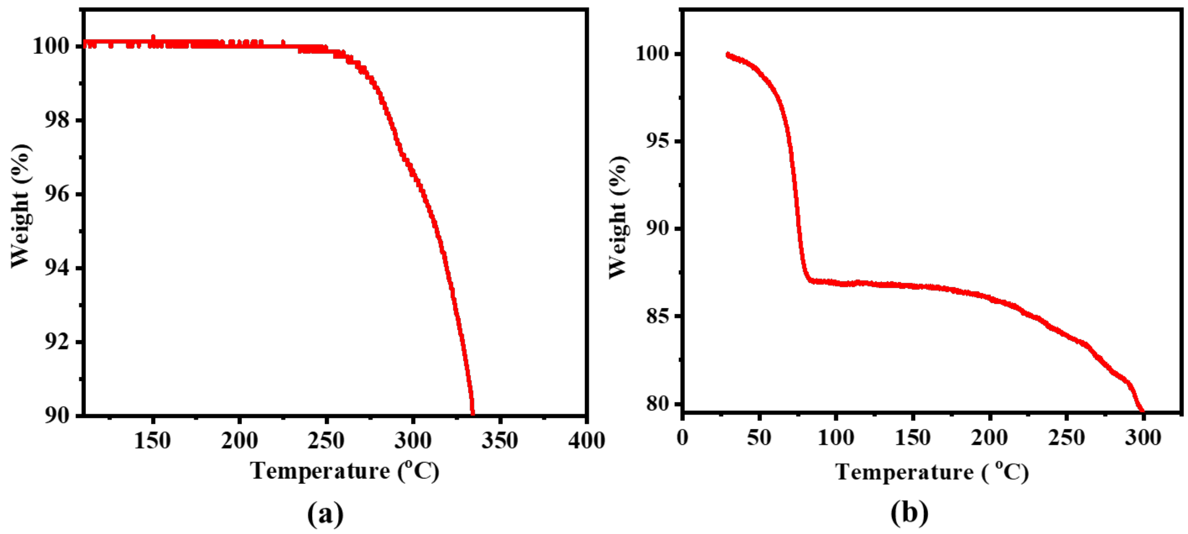

Figure S19. TGA plot, (**a**) for compound M1; (**b**) for DCM treated M1.
